# Genome-wide cell-free DNA methylation analyses improve accuracy of non-invasive diagnostic imaging for early-stage breast cancer

**DOI:** 10.1186/s12943-021-01330-w

**Published:** 2021-02-19

**Authors:** Jiaqi Liu, Hengqiang Zhao, Yukuan Huang, Shouping Xu, Yan Zhou, Wei Zhang, Jiaqi Li, Yue Ming, Xinyu Wang, Sen Zhao, Kai Li, Xiying Dong, Yunlong Ma, Tianyi Qian, Xinyi Chen, Zeyu Xing, Yan Zhang, Hongyan Chen, Zhihua Liu, Da Pang, Meng Zhou, Zhihong Wu, Xiaowo Wang, Xiang Wang, Nan Wu, Jianzhong Su

**Affiliations:** 1 Department of Breast Surgical Oncology, National Cancer Center/National Clinical Research Center for Cancer/Cancer Hospital, Chinese Academy of Medical Sciences and Peking Union Medical College, Beijing, 100021 China; 2 Institute of Biomedical Big Data, Wenzhou Medical University, Wenzhou, 325027 China; 3 Department of Orthopedic Surgery, Beijing Key Laboratory for Genetic Research of Skeletal Deformity & Key Laboratory of Big Data for Spinal Deformities, Peking Union Medical College Hospital, Peking Union Medical College and Chinese Academy of Medical Sciences, Beijing, 100730 China; 4 School of Biomedical Engineering, School of Ophthalmology & Optometry and Eye Hospital, Wenzhou Medical University, Wenzhou, 325027 China; 5 Department of Breast Surgery, Harbin Medical University Cancer Hospital, Harbin, 150081 China; 6 College of Mathematics and Statistics, Institute of Statistical Sciences, Shenzhen University, Shenzhen, 518060 China; 7 Ministry of Education Key Laboratory of Bioinformatics, Center for Synthetic and Systems Biology, Department of Automation, Tsinghua University, Beijing, 100084 China; 8 PET-CT Center, National Cancer Center/National Clinical Research Center for Cancer/Cancer Hospital, Chinese Academy of Medical Sciences and Peking Union Medical College, Beijing, 100021 China; 9 Wenzhou Institute, University of Chinese Academy of Sciences, Wenzhou, 325011 China; 10 School of Life Science and Biotechnology, Harbin Institute of Technology, Harbin, 150001 China; 11 State Key Laboratory of Molecular Oncology, National Cancer Center/National Clinical Research Center for Cancer/Cancer Hospital, Chinese Academy of Medical Sciences and Peking Union Medical College, Beijing, 100021 China; 12 Medical Research Center, State Key Laboratory of Complex Severe and Rare Diseases, Peking Union Medical College Hospital, Peking Union Medical College and Chinese Academy of Medical Sciences, Beijing, 100730 China

## Abstract

Early detection is crucial to improve breast cancer (BC) patients’ outcomes and survival. Mammogram and ultrasound adopting the Breast Imaging Reporting and Data System (BI-RADS) categorization are widely used for BC early detection, while suffering high false-positive rate leading to unnecessary biopsy, especially in BI-RADS category-4 patients. Plasma cell-free DNA (cfDNA) carrying on DNA methylation information has emerged as a non-invasive approach for cancer detection. Here we present a prospective multi-center study with whole-genome bisulfite sequencing data to address the clinical utility of cfDNA methylation markers from 203 female patients with breast lesions suspected for malignancy. The cfDNA is enriched with hypo-methylated genomic regions. A practical computational framework was devised to excavate optimal cfDNA-rich DNA methylation markers, which significantly improved the early diagnosis of BI-RADS category-4 patients (AUC from 0.78–0.79 to 0.93–0.94). As a proof-of-concept study, we performed the first blood-based whole-genome DNA methylation study for detecting early-stage breast cancer from benign tumors at single-base resolution, which suggests that combining the liquid biopsy with the traditional diagnostic imaging can improve the current clinical practice, by reducing the false-positive rate and avoiding unnecessary harms.

**Supplementary Information:**

The online version contains supplementary material available at 10.1186/s12943-021-01330-w.

## Main text

Breast cancer (BC) is the most common cancer in women worldwide [1]. Mammogram and ultrasound are routinely administered to detect early BC in asymptomatic females, but prone to underestimation or over-diagnosis [1, 2]. The Breast Imaging Reporting and Data System (BI-RADS) categories, based on mammograms and ultrasonography, have been used to standardize the risk assessment for breast lesions [3]. However, the risk of malignancy for BI-RADS category 4 lesions varies from 3 to 94%, and this large statistical dispersion might lead to unnecessary biopsies according to current clinical guidelines [4].

Mutation-based circulating tumor DNA (ctDNA) analysis has been used for detecting early relapse, analyzing acquired resistance, and guiding adjuvant therapy in several cancers [5–7]. Moreover, combining liquid biopsy with diagnostic imaging has been recently demonstrated to have better performance [8]. However, the lack of multiple common mutations in BC has limited the sensitivity of mutation-based ctDNA detection [9]. On the other hand, DNA methylation-based markers have been effective for the early detection of many cancer types [10, 11]. However, most methylation-based studies were conducted to detect individuals with cancer among a population of non-conditional healthy controls using the locus-specific or CpG-rich genomic regions technologies [10, 11], methylated DNA immunoprecipitation sequencing (MeDIP-seq) [12], and reduced representation bisulfite sequencing (RRBS) [13]. Genome-wide DNA methylation characteristics of cfDNA between the malignant and benign tumors at single-base resolution remain largely unknown.

### Study design

**Fig. 1:**
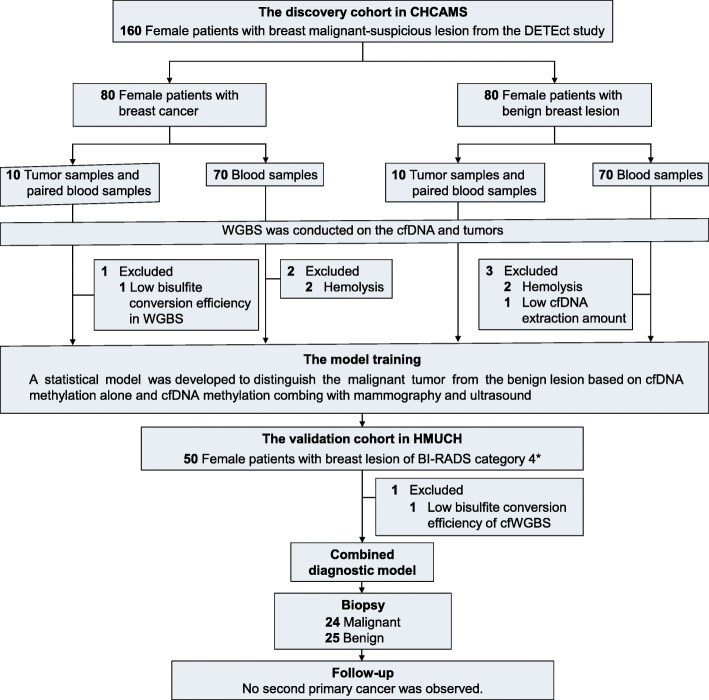
Patient enrollment and sample collection. We recruited 210 consecutive female patients from the Cancer Hospital of the Chinese Academy of Medical Sciences and Peking Union Medical College (CHCAMS, *n* = 160, the discovery cohort) and the Harbin Medical University Cancer Hospital (HMUCH, *n* = 50, the validation cohort) from April 1, 2019, to August 31, 2019, as part of the DETEct study (Deciphering Epigenetic signatures in Tumor and Exploiting ctDNA)

**Fig. 2:**
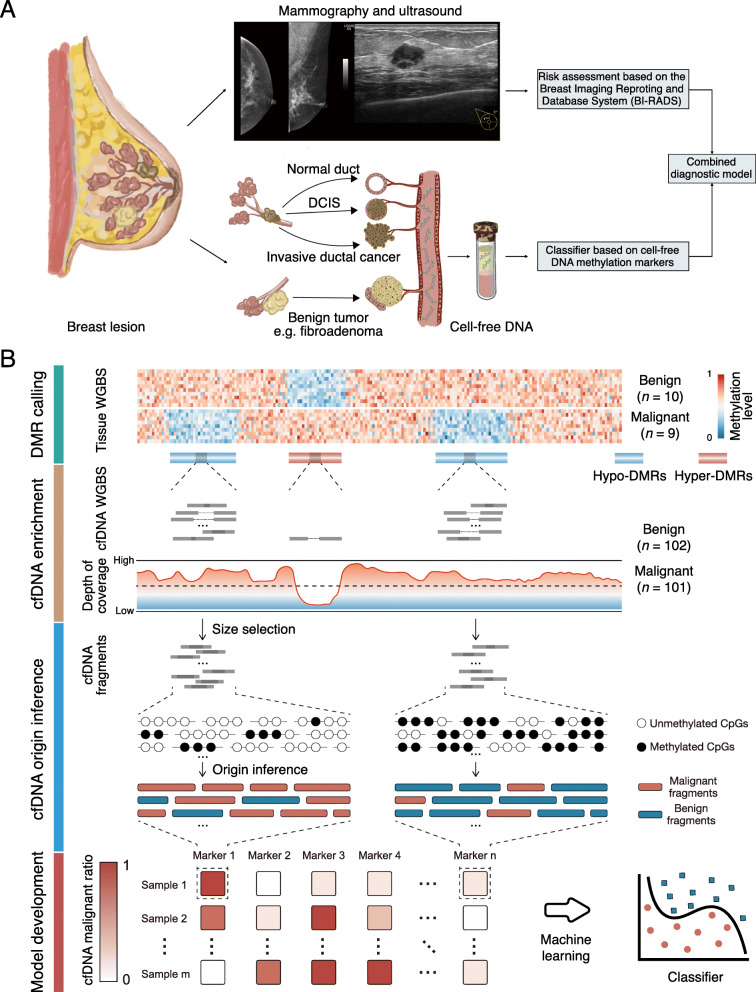
The workflow of data analysis and models development. **a** The images of standard mammography and ultrasonography were interpreted and classified according to the fifth edition of the Breast Imaging Reporting and Database System (BI-RADS) standard by two experienced radiologists independently at each center. The cfDNA methylome of each participant was measured by the whole-genome bisulfite sequencing (WGBS). Additionally, machine learning was applied to identify cfDNA methylation markers of early-stage breast cancers. Finally, a diagnostic model using the identified cfDNA methylation markers in combination with radiology and ultrasound findings was accessed the ability to improve the accuracy of early-stage breast cancer detection. DCIS, ductal carcinoma in situ. **b** A comprehensive framework was devised to develop the classifier for identifying early-stage breast cancer based on the cfDNA methylation markers from massive cfDNA WGBS fragments. It includes four processes: the differentially methylated region (DMR) calling, cfDNA enrichment, cfDNA origin inference, and model development. First, 5613 hyper-DMRs and 51,962 hypo-DMRs were identified from WGBS data of 10 benign and 9 malignant breast primary tissue samples. Second, the cfDNA enrichment scores were computed by the mean number of fragments in DMRs. Then, fragment size selection was conducted to reduce the effect of plenty of non-tumor cfDNA in plasma based on that ctDNA fragments are shorter than non-tumor cfDNA fragments. Next, a fragment-based strategy was devised to statistically infer the origin (malignant or not) of each fragment, based on the distributions of DNA methylation pattern of tissues in DMRs. Finally, a predictive score (cfMeth score) based on cfDNA methylation ratio in each plasma sample was computed using a random forest classifier

Herein, we recruited 210 consecutive female patients with BI-RADS category 4 breast lesions that were biopsied after mammography and ultrasonography examinations from the Cancer Hospital of the Chinese Academy of Medical Sciences and Peking Union Medical College (CHCAMS, *n* = 160, discovery cohort) and the Harbin Medical University Cancer Hospital (HMUCH, *n* = 50, validation cohort) from April 1, 2019, to August 31, 2019. This study was reviewed and approved by the ethics committee of each participating hospital. Each participant provided written informed consent. The diagnosis of each patient was based on the pathology results from resection specimens by the surgical biopsies or core needle biopsies. Twenty tumor samples (10 malignant and 10 benign) were collected for whole-genome bisulfite sequencing (WGBS) from patients that underwent biopsies at CHCAMS. A median of 3 mL of plasma was collected from all participants (*n* = 210) before surgery (Fig. 1). A diagnostic model using the identified cfDNA methylation markers alone or in combination with imaging findings improved the accuracy of early-stage BC detection (Fig. 2a). The cfDNA methylome of each participant was also measured by the WGBS. Because of the low amount of ctDNA in the total cfDNA, we devised a computational framework to boost the detection of cfDNA methylation markers, which consisted of identification of differentially methylated regions (DMRs) based on the tumor samples, cfDNA enrichment analysis, fragment size selection, fragment-based statistical inference for cfDNA malignant ratios, and a prediction model of early-stage BC (Fig. 2b; Supplementary methods).

### Clinical characteristics of the participants

Six patients in the discovery cohort and one patient in the validation cohort were excluded due to low data quality. A total of 77 patients with BCs and 77 patients with benign lesions from CHCAMS constituted the discovery cohort. Forty-nine patients from HMUCH constituted the validation cohort, 24 of whom had biopsy-confirmed BCs (Fig. 1). The breast lesions were further interpreted as BI-RADS subcategories 4a, 4b, and 4c, depending on the probability of malignancy. Patients were followed up every 3 months for 6 months using mammograms and ultrasounds until a final diagnosis was made. All of the patients with confirmed BCs were in the early stages of the disease, and most of them were hormone receptor positive/luminal (Tables S1 and S2).

### Identification of cfDNA methylation markers

The cfDNA concentrations were surveyed in each cohort, and none of them could directly discriminate between malignant and benign tumors (Fig. S1A). The quality and size distribution of plasma DNA samples was assessed by the Agilent 2100 Bioanalyzer (Agilent, USA). All distribution showed a distinct peak for the cfDNA (Fig. S1B). Furthermore, to remove the genomic DNA contamination, fragments lower than 500 bp in length were retained during bead-based library purification. After library preparation and sequencing, we inferred the size profile of cfDNA by analyzing the WGBS data (*n* = 101 malignant and 102 benign), which showed a typical pattern of the size distribution for cfDNA with a prominent mode at 167 bp (Fig. S1C). The cfDNA together with the 19 tissue samples yielded a total of 4.0 Tb of WGBS data, covering roughly 88% of the reference genome with 11.2× depth on average (Table S3).

**Fig. 3:**
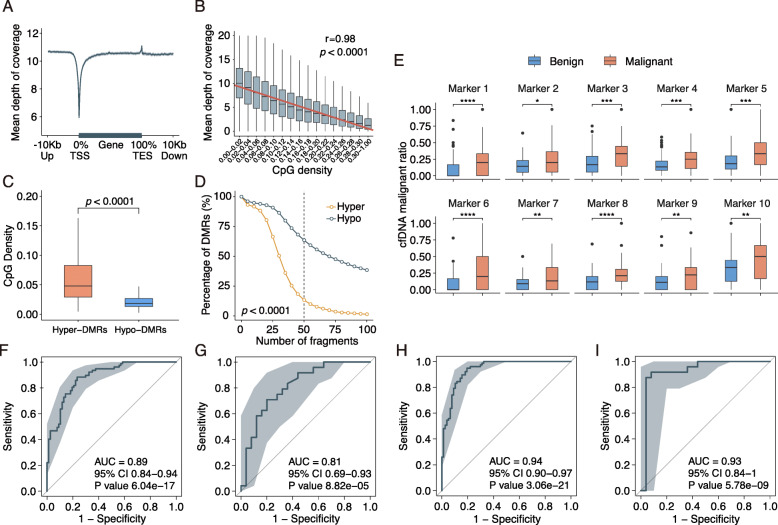
The cell-free DNA methylation landscapes and the cfDNA methylation analysis for breast cancer diagnosis. **a** Gene body with ±10 kb profiles of mean sequencing coverage of cfDNA fragments using WGBS. The gene lengths were normalized to 20 kb. The cfDNA fragments were enriched in coding regions and intergenic regions compared to the loss in gene promoter regions. TSS, transcription start site; TES, transcription end site. **b** The amount of cfDNA in different genomic regions negatively correlating with their CpG density (*r* = 0.98, *p* < 0.0001). **c** Boxplots showing CpG density was significantly higher in hyper-DMRs than in hypo-DMRs (*p* < 0.0001). Boxplots represent the interquartile range (25–75%), with the median; whiskers correspond to 1.5 times the interquartile range. **d** The average amount of cfDNA fragments in hypo-DMRs is significantly higher than ones in hyper-DMRs in all of the malignant and benign samples (*p* < 0.0001 by Pearson’s chi-squared test). **e** The cfDNA malignant ratio of the top 10 optimal cfDNA hypo-DMRs markers in plasma samples from patients with breast cancer and benign breast lesions. ns, not significant; * *p* ≤ 0.05; ** *p* ≤ 0.01; *** *p* ≤ 0.001; **** *p* ≤ 0.0001. **f** and **g** Receiver operating characteristic (ROC) curves of the diagnostic model based on the cfMeth score. The area under the curve (AUC) of the cfMeth score obtained for the discovery (**f**) and validation (**g**) cohorts were 0.89 (95% CI, 0.84–0.94) and 0.81 (95% CI, 0.69–0.93). **h** and **i** ROC curves of the combined diagnostic model. The AUC of the cfMeth score obtained for the discovery (**h**) and validation (**i**) cohorts were 0.94 (95% CI, 0.90–0.97) and 0.93 (95% CI, 0.84–1.00)

We found that cfDNA fragments of WGBS are enriched in coding regions and intergenic regions compared to the loss in gene promoter regions (Fig. 3a). The amount of corresponding cfDNA to different genomic regions was negatively correlated with the CpG density and GC content (Fig. 3b and Fig. S2). A total of 57,575 DMRs (51,962 hypo-DMRs vs. 5613 hyper-DMRs) was identified between 9 malignant and 10 benign tumor samples (Fig. S3). As expected, CpG density was significantly higher in hyper-DMRs than in hypo-DMRs (*p* < 0.0001; Fig. 3c). Accordingly, the average amount of cfDNA fragments in hypo-DMRs is significantly higher than ones in hyper-DMRs in all of malignant and benign samples (*p* < 0.0001; Fig. 3d). The depletion of the cfDNA fragments in CpG-rich hyper-DMRs could be due to the preferential digestion of open chromatin regions in cfDNA [14]. Those findings help us to optimize cfDNA methylation markers in hypomethylated regions. Aberrant hypomethylation, generally in the closed chromatins of cfDNA-rich gene bodies or intergenic regions, is a common feature of various malignant tumors [14]. To ensure quantification of high-quality cfDNA, the hypo-DMRs were selected as candidate DNA methylation markers.

The cfDNA malignant ratio was computed for the hypo-DMRs for each patient sample (Supplementary methods). The 10 optimal hypo-DMRs to distinguish between malignant and benign plasma samples were selected using data from the discovery cohort. They were mostly in the intergenic regions, in which the malignant ratios were significantly higher from patients with malignant tumors than ones from patients with benign lesions (*p* < 0.05 for all; Fig. 3e). However, there are four functional genes (*RYR2*, *RYR3*, *GABRB3*, and *DCDC2C*) and two lncRNAs (AC096570.1 and LINC00923) in the ten methylation markers (Table S4).

### A prediction model for early-stage breast cancer using cfDNA methylation

Using the cfDNA malignant ratios of the markers, a predictive score of cfDNA methylation (cfMeth score) in each plasma sample in the discovery cohort was computed using a random forest classifier. To reduce overfitting, 10-fold cross-validation was used (Fig. S4). The area under the curve (AUC) of the model obtained for the discovery cohort and the validation cohort were 0.89 (95% CI, 0.84–0.94; Fig. 3f) and 0.81 (95% CI, 0.69–0.93; Fig. 3g), respectively, which was superior to the AUCs of BI-RADS findings (AUC = 0.78–0.79 for mammography and ultrasound) or relevant tumor biomarkers (AUC = 0.57–0.70 for CA15–3 and CEA; Fig. S5). The cfMeth scores in the prediction model were positively and robustly associated with the stages of BC, which include the ductal carcinoma in situ (Fig. S6). Additionally, lower cfMeth scores were associated with lower histologic grade and proliferation indices (Ki-67 measured by immunohistochemistry; Figs. S6 and S7).

### A combinational model of cfMeth scores and diagnostic imaging

A diagnostic model combining cfMeth scores with mammography and ultrasound was developed in the discovery cohort using the ridge logistic regression. The combined model performed better than either one of the separate approaches (AUC = 0.94, 95% CI, 0.90–0.97; Fig. 3h). The malignant and benign groups had statistically different distributions of the combined scores in both the discovery and validation cohorts (Fig. S8). A cutoff point of the combined model was selected to be at which the false-negative rate was less than 2% in the discovery cohort, with a sensitivity of 98.7%, a specificity of 68.8%, and an accuracy of 83.8% (Table S6). In the validation cohort, the performance was similar (AUC = 0.93 [95% CI, 0.84–1.00]; Fig. 3i) with a sensitivity of 91.7%, a specificity of 88.0%, and an accuracy of 89.8%, which demonstrated no evidence of overfitting. Overall, the detection rate of the combined model was 93.3% (42/45), 100% (34/34), and 100% (22/22) at a specificity of 73.5% for stage I, II, and III, respectively (Table S5).

## Discussion

Liquid biopsies are emerging as a non-invasive adjunct or alternative to standard tumor biopsies [5–7, 10, 11]. Compared to previous locus-specific methylation analysis [10–13], this study provided a useful resource of extensive data to uncover cfDNA methylation characteristics at the genome-wide scale in breast cancer. Based on the WGBS methods covering ~ 88% human genomes, we have demonstrated the amount of cfDNA in different genomic regions negatively correlating with their CpG density.

Comparing to the recent study of the cfDNA methylation-based classifier for identifying BCs from healthy female controls [15], this study demonstrated a better performance by this combination strategy, even successful at distinguishing malignant breast lesions from benign ones, especially in stage I and II. Clinical use of the combined approach might reduce the number of unnecessary biopsies in women with BI-RADS category 4 findings.

However, there were also some limitations in this study. As a prior study, the tissue sample size was relatively small. Besides, the onset ages were not adequately matched in this study, which might lead to some potential bias in the methylation selection. The large-scale replication studies with more tumor tissue and cfDNA from multicenter will benefit to investigate the subtype-specific methylation biomarkers, the clinical utility and stability of the combined non-invasive strategy in future work.

## Conclusions

In conclusion, we performed a blood-based whole-genome DNA methylation study at the single-base resolution for detecting early-stage breast cancer, which suggests that combining liquid biopsy with traditional diagnostic imaging can improve the accuracy of early-stage breast cancer diagnoses.

## Supplementary Information

**Additional file 1.**

## Data Availability

The supplement data that support the findings of this study are openly available in the supplementary materials. All data can be viewed in NODE (http://www.biosino.org/node) by pasting the accession (OEP000860) into the text search box or through the URL: http://www.biosino.org/node/project/detail/OEP000860. Scripts used to generate the findings in this study have been deposited on https://bitbucket.org/ibbd_wmu/detect-study/src/master/.

## Abbreviations

BC: Breast cancer
BI-RADS: Breast imaging reporting and data system
ctDNA: Circulating tumor DNA
cfDNA: Cell-free DNA
MeDIP-seq: Methylated DNA immunoprecipitation sequencing
RRBS: Reduced representation bisulfite sequencing
WGBS: Whole-genome bisulfite sequencing
DMR: Differentially methylated regions
AUC: Area under the curve
ROC: Receiver operating characteristics

## References

[1] Waks AG, Winer EP (2019). Breast cancer treatment: a review. JAMA.

[2] Ohuchi N, Suzuki A, Sobue T, Kawai M, Yamamoto S, Zheng Y-F, Shiono YN, Saito H, Kuriyama S, Tohno E (2016). Sensitivity and specificity of mammography and adjunctive ultrasonography to screen for breast cancer in the Japan Strategic Anti-cancer Randomized Trial (J-START): a randomised controlled trial. Lancet.

[3] American College of Radiology (2013). American College of Radiology Breast Imaging Reporting and Data System Atlas (BI-RADS Atlas).

[4] Bevers TB, Helvie M, Bonaccio E, Calhoun KE, Daly MB, Farrar WB, Garber JE, Gray R, Greenberg CC, Greenup R (2018). Breast cancer screening and diagnosis, version 3.2018, NCCN clinical practice guidelines in oncology. J Natl Compr Cancer Netw.

[5] Rothwell DG, Ayub M, Cook N, Thistlethwaite F, Carter L, Dean E, Smith N, Villa S, Dransfield J, Clipson A (2019). Utility of ctDNA to support patient selection for early phase clinical trials: the TARGET study. Nat Med.

[6] Ye Q, Ling S, Zheng S, Xu X (2019). Liquid biopsy in hepatocellular carcinoma: circulating tumor cells and circulating tumor DNA. Mol Cancer.

[7] Lim SY, Lee JH, Diefenbach RJ, Kefford RF, Rizos H (2018). Liquid biomarkers in melanoma: detection and discovery. Mol Cancer.

[8] Lennon AM, Buchanan AH, Kinde I, Warren A, Honushefsky A, Cohain AT, Ledbetter DH, Sanfilippo F, Sheridan K, Rosica D (2020). Feasibility of blood testing combined with PET-CT to screen for cancer and guide intervention. Science.

[9] Cohen JD, Li L, Wang Y, Thoburn C, Afsari B, Danilova L, Douville C, Javed AA, Wong F, Mattox A (2018). Detection and localization of surgically resectable cancers with a multi-analyte blood test. Science.

[10] Xu RH, Wei W, Krawczyk M, Wang W, Luo H, Flagg K, Yi S, Shi W, Quan Q, Li K (2017). Circulating tumour DNA methylation markers for diagnosis and prognosis of hepatocellular carcinoma. Nat Mater.

[11] Luo H, Zhao Q, Wei W, Zheng L, Yi S, Li G, Wang W, Sheng H, Pu H, Mo H, et al. Circulating tumor DNA methylation profiles enable early diagnosis, prognosis prediction, and screening for colorectal cancer. Sci Transl Med. 2020;12:eaax7533. 10.1126/scitranslmed.aax7533 31894106

[12] Shen SY, Singhania R, Fehringer G, Chakravarthy A, Roehrl MHA, Chadwick D, Zuzarte PC, Borgida A, Wang TT, Li T (2018). Sensitive tumour detection and classification using plasma cell-free DNA methylomes. Nature.

[13] Guo S, Diep D, Plongthongkum N, Fung HL, Zhang K, Zhang K (2017). Identification of methylation haplotype blocks aids in deconvolution of heterogeneous tissue samples and tumor tissue-of-origin mapping from plasma DNA. Nat Genet.

[14] Snyder Matthew W, Kircher M, Hill Andrew J, Daza Riza M, Shendure J (2016). Cell-free DNA comprises an in vivo nucleosome footprint that informs its tissues-of-origin. Cell.

[15] Liu MC, Oxnard GR, Klein EA, Swanton C, Seiden MV, Cummings SR, Absalan F, Alexander G, Allen B, Amini H (2020). Sensitive and specific multi-cancer detection and localization using methylation signatures in cell-free DNA. Ann Oncol.

