# Supplementary material for: Genome-wide cell-free DNA methylation analyses improve accuracy of non-invasive diagnostic imaging for early-stage breast cancer
Source: Mol Cancer. 2021 Feb 19;20:36. doi: 10.1186/s12943-021-01330-w (PMC7893735; doi:10.1186/s12943-021-01330-w)
Supplement: Supplementary file 1 — Additional file 1. [file 12943_2021_1330_MOESM1_ESM.pdf]

Supplementary Materials

Genome-wide Cell-Free DNA Methylation Analyses Improve Accuracy of Non-invasive Diagnostic Imaging for Early-stage Breast Cancer

Liu et al.

Materials and Methods

Fig. S1. Mean cfDNA concentration and fragment size distribution in the plasma of breast cancer patients and patients with breast benign lesions.

Fig. S2. The amount of cfDNA in different genomic regions negatively correlating with their GC content.

Fig. S3. The highly consistent methylation patterns of hypo-DMRs between malignant (n = 9) and benign (n = 10) tumor tissues.

Fig. S4. Model optimization using a grid search technique with 10-fold cross-validation.

Fig. S5. Receiver operating characteristic (ROC) curves of the diagnostic prediction model using mammography, ultrasound, CEA, and CA15-3.

Fig. S6. Evaluating cfMeth score in categories of the Breast Imaging Reporting and Data System (BI-RADS) and with the clinical and pathologic characteristics.

Fig. S7. The cfMeth score correlates ki-67, tumor size, estrogen receptor (ER) and progesterone receptor (PR).

Fig. S8. The distributions of the combined scores in the patients with breast cancer and patients with benign breast lesions in the discovery cohort and the validation cohort.

Table S1. Clinical characteristics of patients in the discovery and validation cohorts.

Table S2. Summary of patients' clinical information in discovery and validation cohorts.

Table S3. Summary of samples and WGBS information.

Table S4. Methylation markers in the diagnostic model.

Table S5. Predictive accuracy of the cell-free DNA methylation analysis (the cfMeth score) combined with mammography and ultrasound in the discovery and validation cohorts.

Table S6. Breast cancer detection rate through the combined score in clinical characteristics at a specificity of 68.8%-88.0%.

This supplementary material has been provided by the authors to give readers additional information about their work.

Materials and Methods

Study Design and Participants

We have recruited 210 consecutive female patients from the Cancer Hospital of the Chinese Academy of Medical Sciences and Peking Union Medical College (CHCAMS, n=160, the discovery cohort) and the Harbin Medical University Cancer Hospital (HMUCH, n=50, the validation cohort) from April 1, 2019, to August 31, 2019, as part of the DETEct study (Deciphering Epigenetic signatures in Tumor and Exploiting ctDNA; Chinese Clinical Trial Registry number: ChiCTR1900026080). Standard mammography and ultrasonography techniques were conducted at two centers. The images were interpreted and classified according to the fifth edition of the BI-RADS standard by two experienced radiologists independently at each center [1]. All participants had BI-RADS category 4 breast lesions that were biopsied after mammography and ultrasonography examinations. This study followed the criteria of REMARK (REporting recommendations for tumor MARKer prognostic studies) [2] and was reviewed and approved by the ethics committee of each participating hospital. Each participant provided written informed consent.

Tumor Sample Collection and DNA Extraction

Tumor biopsies were collected from 20 patients including 10 patients with malignant tumors and 10 with breast benign tumors during the surgery from the Cancer Hospital of the Chinese Academy of Medical Sciences (CHCAMS). The histologic type of tumors from each patient was confirmed with pathology results of hematoxylin and eosin staining. Genomic

DNA was extracted from the fresh-frozen tumor tissue with the QIAamp DNA Mini Kit (QIAGEN, Germany).

Blood Collection and Plasma Isolation

A median of 7 mL (interquartile range, 6-9 mL) of peripheral blood was collected from all participants (n=210) before surgery at the CHCAMS (n=160) and the Harbin Medical University Cancer Hospital (HMUCH, n=50) and stored in 10 ml CELL-FREE DNA BCT® blood collection tubes (Streck, USA) at room temperature (RT, 15-25℃). Plasma was extracted within 48 hours following blood collection by centrifugation of the blood at 1800g for 10 minutes at RT.

Cell-Free DNA Extraction and Quantification

The cfDNA was extracted from plasma using the QIAmp Circulating Nucleic Acid Kit (Qiagen, USA) with the Qiagen QIAvac 24 Plus vacuum manifold and QIAvac Connecting System (Qiagen, USA) according to the manufacturer's recommendations. Then cfDNA was quantified by Qubit 3.0 using the dsDNA HS Assay Kit (Life Technologies, USA). The quality and size distribution of plasma DNA samples was assessed by the Agilent 2100 Bioanalyzer (Agilent, USA). A DNA ladder was used for reference. All distribution showed a well identification of the lower / upper markers and a distinct peak for the cfDNA (~167 bp). At last, DNA was stored at -80℃ for further analysis.

Genomic DNA methylation library preparation.

Genomic DNA and unmethylated lambda DNA (Promega, USA) was sonicated into ~350 bp fragments with the Covaris S220 instrument (Covaris, USA). Then a mixture of genomic

DNA (200ng) and 0.5% unmethylated lambda DNA was prepared and modified with EZ DNA Methylation-Lightning Kit (Zymo Research, USA) and processed into library construction with Accel-NGS Methyl-Seq DNA Library Kit and Methyl-Seq Dual Indexing Kit (Swift Biosciences, USA) according to the manufacturer's protocol.

Briefly, adaptase was applied to the bisulfite-converted DNA, with a low-complexity tail as well as the first truncated adaptors simultaneously ligated to the 3' end of the DNA fragments. Within primer extension and ligation processed, the DNA with truncated adaptors was incorporated, extended with adaptor sequences, and ligated with the second adaptor. Subsequently, DNA fragments with adaptors ligated were purified and enriched by indexing Polymerase Chain Reaction (PCR) according to instruction manuals.

Cell-free DNA Methylation Library Preparation.

To remove the genomic DNA contamination, fragments larger than 500 bp in length were removed during bead-based library purification using the SPRIselect beads (Beckman Coulter, USA). Cell-free DNA (10-50ng) was spiked with 0.5% unmethylated lambda DNA (Promega, USA) and subjected to bisulfite conversion with EZ DNA Methylation-Lightning Kit (Zymo Research, USA). The converted DNA was processed into library preparation with Accel-NGS Methyl-Seq DNA Library Kit and Methyl-Seq Dual Indexing Kit (Swift Biosciences, USA) according to the manufacturer's protocol.

Library Quantification and Whole Genome Bisulfite Sequencing (WGBS).

The Prepared libraries were quantified with Qubit dsDNA HS Assay Kit (Life Technologies, USA) and KAPA Library Quantification Kit (KAPA Biosystems, USA), and the

library quality was assessed using Agilent 2100 Bioanalyzer (Agilent, USA). Paired-end 150 bp sequencing was performed for each library on the Illumina HiSeq platform to a mean coverage depth of 10X for both the genomic DNA and cfDNA.

Quality Control, Data processing, and analysis

Quality control (QC) analysis was performed to assess read quality of WGBS using FastQC (version 0.11.8, www.bioinformatics.babraham.ac.uk/projects/fastqc/). The raw sequencing reads was processed to remove adapter contamination and filter out poor quality reads using trim_galore (version 0.6.0, www.bioinformatics.babraham.ac.uk/projects/trim_galore/). The sequencing reads were mapped using Bismark (version 0.22.1). [3, 4] The variant-calling and annotation were performed by the in-house developed PUMP (Peking Union Medical college hospital Pipeline).[5, 6] All of CpG sites with variants were removed. The methylation status of every single C site in the CpG context was extracted for each read using Bismark methylation extractor script and merged reads on both strands of a CpG dinucleotide. For tissue samples, the methylation level of a CpG site was calculated by the script “methylation extractor” of Bismark. For plasma samples, the methylation status of individual CpG in each read was retained for further analysis. And Samtools suite (version 1.9) was used to manipulate alignments in the BAM format [7]. We used bedtools utilities (version 2.29.0) for the comparison, manipulation and annotation of genomic features in Browser Extensible Data (BED).[8]

Enhanced Detection of ctDNA by Fragment Size Selection

Previous studies revealed that ctDNA fragments are shorter than non-tumor cfDNA fragments. Thus, the shorter fragments were selected to enhance the detection of tumor signal. For thresholds selection, we performed a comprehensive comparison of prediction accuracy on different fragment size selection strategies (all fragments; fragments with length < 160 bp; fragments with length < 155 bp; fragments with length < 150 bp). Based on the different thresholds of the fragment sizes, we assessed the performance of prediction accuracy in discovery cohort as described in our computational framework, using the 10-fold cross-validated random forest. Removing the fragments with the length of more than 160 bp achieved the high prediction accuracy. Therefore, the shorter fragments (<160 bp), accounting for ~30% of the total cfDNA fragments, were kept for further analysis in this study.

Algorithms for identification of cfDNA methylation markers and machine learning

A comprehensive framework was devised to identify optimal cfDNA methylation markers for distinguishing between benign and malignant samples from blood-based WGBS data. It includes several steps, considering the breast tumor tissues of origin, cfDNA fragment enrichment, fragment size selection, cfDNA malignant ratio inference, and optimal marker selection. The details of the discovery set and the validation set were shown in Table S1.

Identification of differentially methylated regions from primary tumor tissues

We identified the differentially methylated regions (DMRs) from WGBS data of 10 benign and 9 malignant breast primary tissue samples using our previously developed tool SMART2 [9, 10]. The differentially methylated regions between malignant and benign tumors samples

were identified with the rigid thresholds at least including 5 CpG sites, the two-sample t-test p value < 0.001, length larger than 500 bp and absolute DNA methylation difference level > 0.2.

cfDNA enrichment analysis

We performed genome-wide metagene enrichment analyses of cfDNA using Refseq gene annotation in the UCSC table browser. Each gene was normalized into 20 kb and the flanking 10kb regions were split into 40 bins with a 100 bp window. cfDNA enrichment scores were computed by the mean number of fragments in DMRs using cfDNA WGBS data. For each sample, the sum read number was normalized to 0.25 billion. For DNA sequence features analysis, the human genome was split into about 3 million bins with 1kb. The CpG density, G+C content, and cfDNA enrichment scores were computed at each bin. Linear regression analysis was performed to assess the correlation between the mean depth of coverage and CpG density (G+C content) of each bin.

Statistical inference for cfDNA malignant ratio based on cfDNA methylation fragments

The content of ctDNA is still low even in shorter cfDNA in theory. The traditional methods for DMRs using the average methylation level difference are masked by a high proportion of the non-tumor cfDNA in plasma. A fragment-based strategy was devised to statistically infer the origin (malignant or not) of each fragment, based on the distributions of DNA methylation pattern of tissues in DMRs. Here, we employed a population diagonal quadratic discriminant analysis (DQDA) method [11] to identify malignant tumor origin of each fragment in interested regions at single-base resolution. The DQDA is derived from the Bayes rule, that is

$$P(y^* = k|\boldsymbol{x}^*) \propto f_k(\boldsymbol{x}^*)\pi_k \quad , \qquad (1)$$

where y^* indicates the class label (k=0, benign or 1, malignant) of each fragment, $\boldsymbol{x}^*$ represents the vector of all CpG methylation states in one cfDNA fragment, f_k is the probability density function of $\boldsymbol{x}^*$ in class k, and π_k is the prior probability that the fragment comes from class k. The decision rule is to assign $\boldsymbol{x}^*$ to the class with a label $\underset{k}{\text{argmin}}\, d_k^Q(\boldsymbol{x}^*)$ under the assumption of the different covariance matrix for a different set, where $d_k^Q(\boldsymbol{x}^*)$ is the discriminant score defined as

$$d_k^Q(\boldsymbol{x}^*) = (\boldsymbol{x}^* - \mu_k)^{\mathrm{T}}\Sigma^{-1}(\boldsymbol{x}^* - \mu_k) + \ln|\Sigma_k| - 2\ln\pi_k. \qquad (2)$$

Note that the population parameters in the score just provided are unknown and need to be estimated from the sample data. Here, let $D_k = \text{diag}(s_{k1}^2, \cdots, s_{kP}^2)$ be the diagonal matrix of the sample covariance matrix. Dudoit et al.[11] replaced μ_k and Σ_k by $\bar{x}_k$ and D_k in (1) and formed the DQDA,

$$\hat{d}_k^Q(\boldsymbol{x}^*) = \sum_{p=1}^{P}\left(x_p^* - \bar{x}_{kp}\right)^2/s_{kp}^2 + \sum_{p=1}^{P}\ln s_{kp}^2 - 2\ln\pi_k, \qquad (3)$$

where $\boldsymbol{x}^* = (x_1^*, \cdots, x_P^*)$ is a given fragment with P CpG sites, $k = 0, 1$ represents benign and malignant sample set respectively, $s_{kp}^2 = \sum_{i=1}^{n_k}\left(x_{kpi} - \bar{x}_{kp}\right)^2 / \ (n_k - 1)$ and $\bar{x}_{kp} = \sum_{i=1}^{n_k} x_{kpi}/n_k$ is the sample means of the ith CpG site in kth group, where n_k is the number of tissue samples in each group. Considering only a small amount of cfDNA fragments should originate from malignant tissue, we estimated the prior probability π_k within the training data as 0.1. After assessing the DQDA score between a given fragment and benign/malignant reference with the above formula,

$y_i = \underset{k}{\operatorname{argmin}}\, \hat{d}_k^Q(\boldsymbol{x}^*)$ was used to infer the source of the fragment. And cfDNA malignant ratio of the given region in each sample was calculated as follows,

$$\text{cfDNA malignant ratio} = \frac{1}{N}\sum_{i=1}^{N} y_i{}^*, \qquad (4)$$

where $y_i{}^*$ represents the class label of the ith fragment in the given region, and N represents the total number of cfDNA fragments tested in the given region.

Feature selection and classifier development

To identify the optimal cfDNA methylation markers for distinguishing malignant samples from benign samples, we calculated the malignancy ratio of each sample in those hypomethylated regions using a 1-kb sliding window. And the optimal 10 features were selected by the recursive feature elimination (RFE) strategy[12] for the classifier development using the random forest algorithm. To reduce overfitting, we used 10-fold CV to tune the main parameters (mtry and nodesize) in the random forest model. Parameter mtry refers to the number of features we should pick for splitting at each tree node. And parameter nodesize refers to the minimum sample size of terminal nodes. By the grid search technique, every combination of parameters was used to evaluate the prediction accuracy of 10-fold CV to find the optimal mtry value and nodesize value. After tuning, we found that mtry = 2 and nodesize = 2 were the best parameter combination for our model and could achieve the highest accuracy (Supplementary Fig. S4).

Clinical Evaluation

We conducted phenotypic analyses of the age, imaging manifestation, pathology features, molecular subtype, stage, and serum tumor markers such as carcinoembryonic antigen (CEA) and cancer antigen 15-3 (CA15-3) in the two cohorts. CEA and CA15-3 analysis were performed by each hospital. The threshold levels were set to 5.0 ng/mL and 25.0 U/mL for CEA and CA15-3, respectively. The diagnosis of each patient was based on the pathology results from resection specimens. Hormone Receptor (HR, including estrogen receptor and progesterone receptor) positive as defined as more than 1% of tumor cells stain positive for estrogen receptor or progesterone receptor proteins. ERBB2/HER2 positive was defined as tumor cells stain strongly (3+) for ERBB2 protein or ERBB2 gene is amplified in tumor cells. Triple-negative was defined as the tumor that does not meet any pathologic criteria for the positivity of estrogen receptor, progesterone receptor, or ERBB2/HER2.[13] Clinical grouping of molecular subtypes were defined by the status of hormone receptor and HER2 according to the St. Gallen 2017 criteria [14]. The staging was determined by the status of the primary tumor (T), lymph node (N), and metastasis (M) according to the eighth edition of classification for breast cancer of the American Joint Commission of Cancer (AJCC) [15].

Statistical Analysis

The student’s t-test was used to analyze the participants’ age, cfDNA concentrations, and differences in cfDNA methylation markers. Pearson's chi-squared test was used to test the difference in hyper-DMR and hypo-DMR enrichment in cfDNA. The sensitivity, specificity, accuracy, and area under the curves (AUCs) with receiver operating characteristics (ROC) were calculated to evaluate the diagnostic performance of the cfDNA methylation markers

alone and in combination with mammography and ultrasonography findings. Statistical analysis was performed using the R statistical software, version 3.5.1.

Sample Size Estimation

In this study, the sample sizes were decided to identify the differential cfDNA malignant ratio in the discovery cohort and to verify that the AUC is non-inferior to 0.80 in the independent validation cohort, respectively. In the discovery cohort, the standard deviations of the cfDNA malignant ratios were 0.226 in the malignant group and 0.214 in the benign group. Thus, the minimum sample size of 39 in each group could achieve a two-sided 95% confidence interval with a distance from the difference in means no less than 0.10 [16, 17]. To ensure the statistic power, we recruited 80 female patients with benign breast lesions and 80 female patients with breast cancers to construct the discovery cohort from the CHCAMS. On the other hand, the minimum sample size was 23 for both malignant and benign tumor patients in the independent validation cohort to effectively verify that the performance (AUCs) of the combined model is non-inferior to the performance of traditional diagnostic imaging with the power (1-Beta) of 82% and significance level (Alpha) of 0.05 using a one-sided z-test [18, 19]. Thus, we finally enrolled 49 patients including 24 patients with benign breast lesions and 25 patients with breast cancers in the independent validation cohort from the HMUCH. The sample size estimation was conducted by the PASS 11.0 (UCSS, USA).

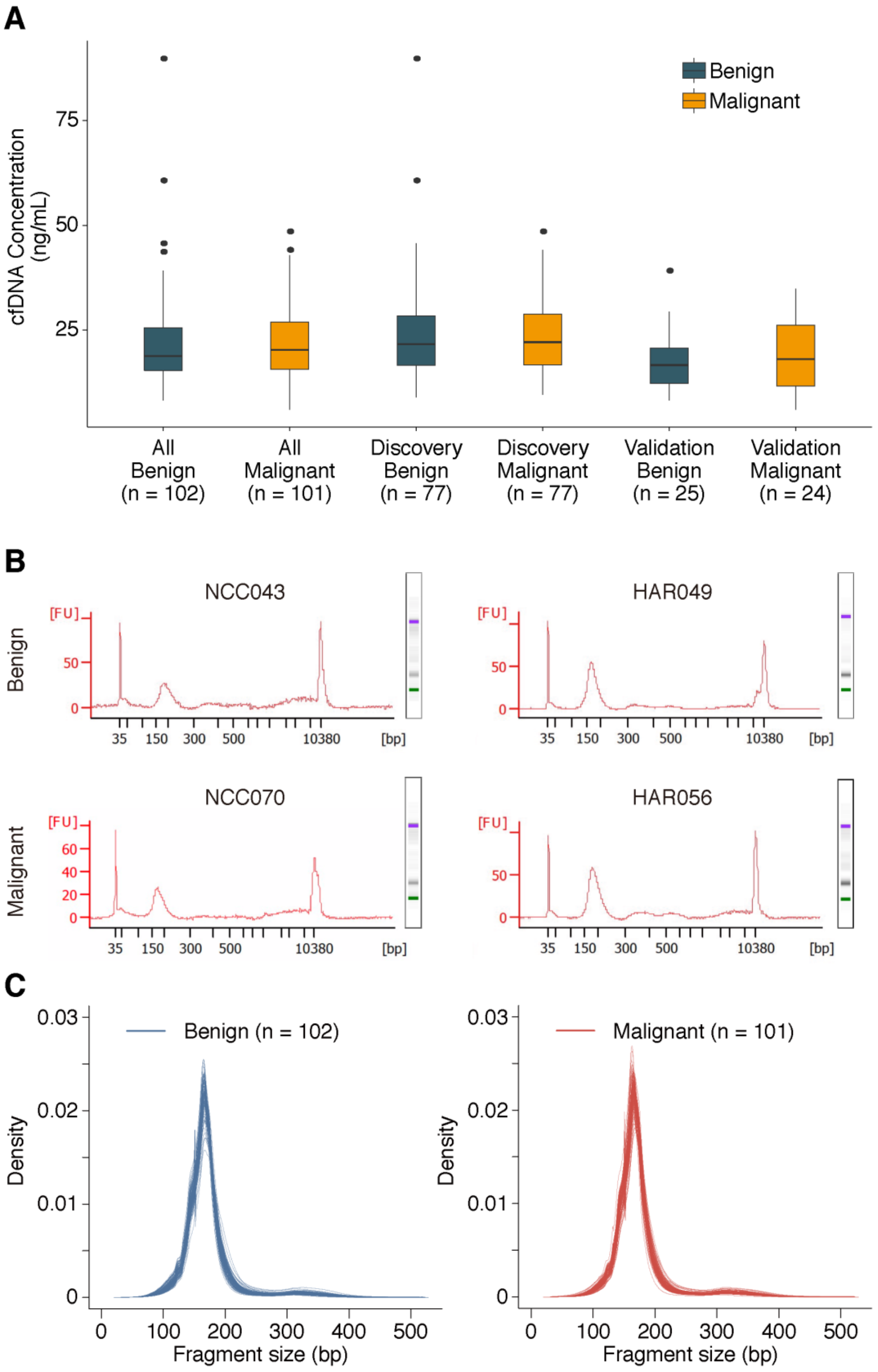
A
Benign
Malignant
cfDNA Concentration (ng/mL)
75
50
25
All Benign (n = 102)
All Malignant (n = 101)
Discovery Benign (n = 77)
Discovery Malignant (n = 77)
Validation Benign (n = 25)
Validation Malignant (n = 24)
B
NCC043
HAR049
NCC070
HAR056
Benign
Malignant
[FU]
[bp]
35
150
300
500
10380
C
Benign (n = 102)
Malignant (n = 101)
Density
Fragment size (bp)

Fig. S1. Mean cfDNA concentration and fragment size distribution in the plasma of breast cancer patients and patients with breast benign lesions. (A), The mean cfDNA concentration could not discriminate between malignant and benign tumors. **(B)**, The quality and size distribution of plasma DNA samples was assessed by the Agilent 2100 Bioanalyzer (Agilent, USA). A DNA ladder was used for reference. All distribution showed a clear identification of the lower marker (35 bp; green ladder), upper marker (10380 bp; purple ladder), and a distinct peak for the cfDNA. **(C)**, To remove the genomic DNA contamination, fragments larger than 500 bp in length were removed during bead-based library purification. The fragment size profile of cfDNA was measured by paired-end sequencing of plasma of patients with breast benign lesions and breast cancers, and it showed a typical pattern of the size distribution for cfDNA, with a prominent mode at 167 bp, which is the length of DNA wrapped around the chromatosome (nucleosome and linker histone), also indicated that there was no difference in the fragment size distribution in cfDNA isolated from plasma of two groups of patients.

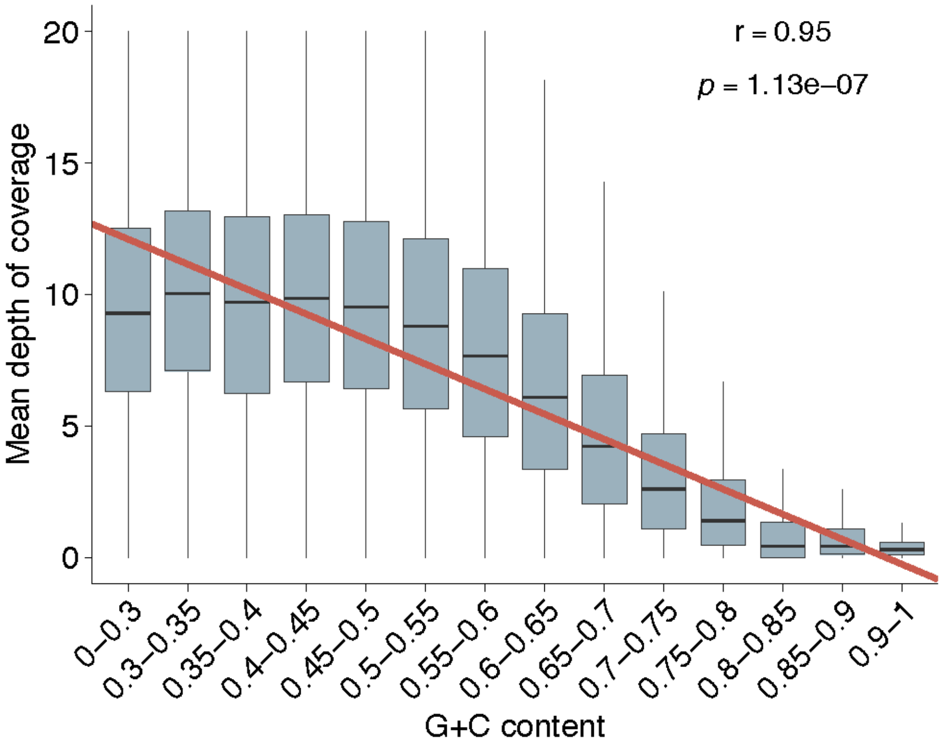

Fig. S2. The amount of cfDNA in different genomic regions negatively correlating with their GC content. The human genome was split into about 3 million bins with 1 kb. The bins were grouped by their GC content. Linear regression analysis was performed to assess the correlation and indicated a negative correlation between the mean depth of coverage and GC content of each group ($r=0.95$, $p=1.13\times10^{-7}$). CpG density is defined as the ratio of CpG sites within a 1-kb bin. GC content is defined as the ratio of G and C sites within a 1-kb bin.

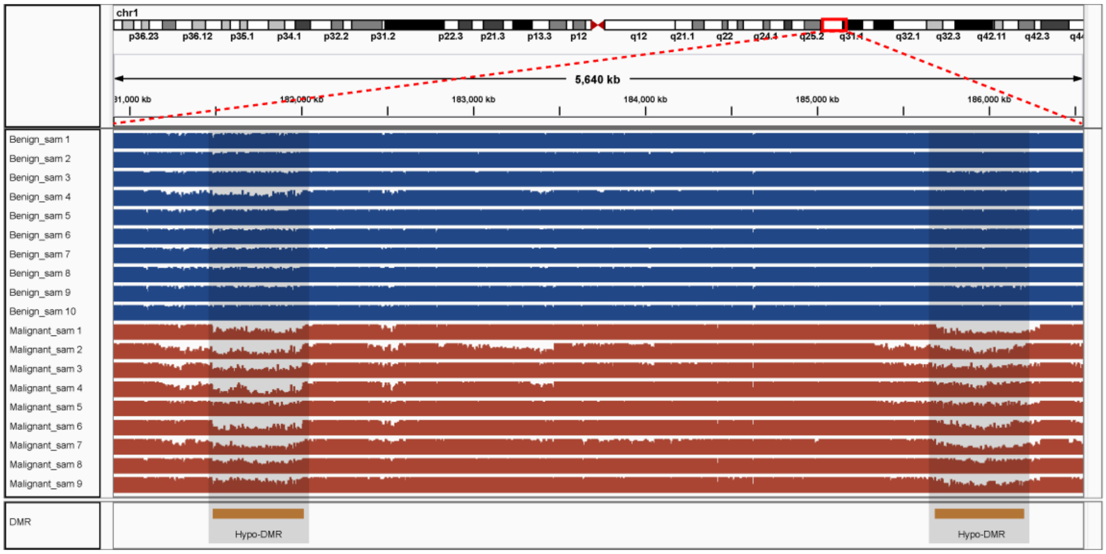

Fig. S3. The highly consistent methylation patterns of hypo-DMRs between malignant (n = 9) and benign (n = 10) tumor tissues. Illustration of methylation patterns of two hypo-DMRs using Integrative Genomics Viewer [20]. Each track represents one sample, and the height of the track represents a methylation ratio ranging from 0 to 1.

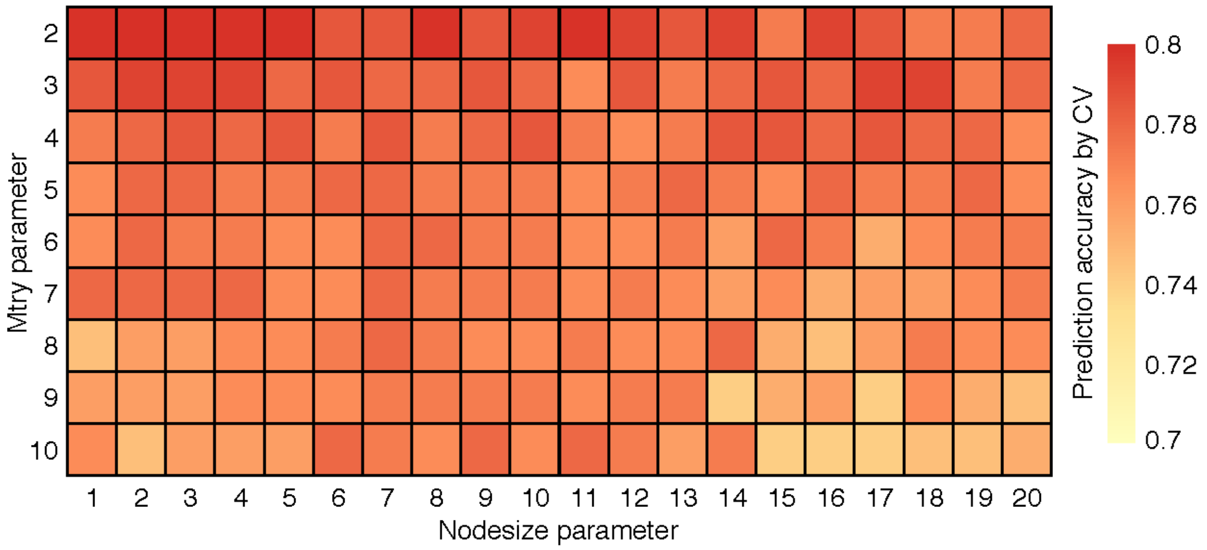

Fig. S4. Model optimization using a grid search technique with 10-fold cross-validation. To reduce overfitting, 10-fold cross-validation was performed to tune the main parameters (mtry and nodesize) of the random forest model using a grid search technique. The X-axis of the grid shows the range of parameter mtry (from 2 to 10), which refers to the number of features we should pick for splitting at each tree node and the Y-axis shows the range of parameter nodesize (from 1 to 20), which refers to the minimum sample size of terminal nodes. The color of the grid represents prediction accuracy using each combination of parameters with 10-fold cross-validation.

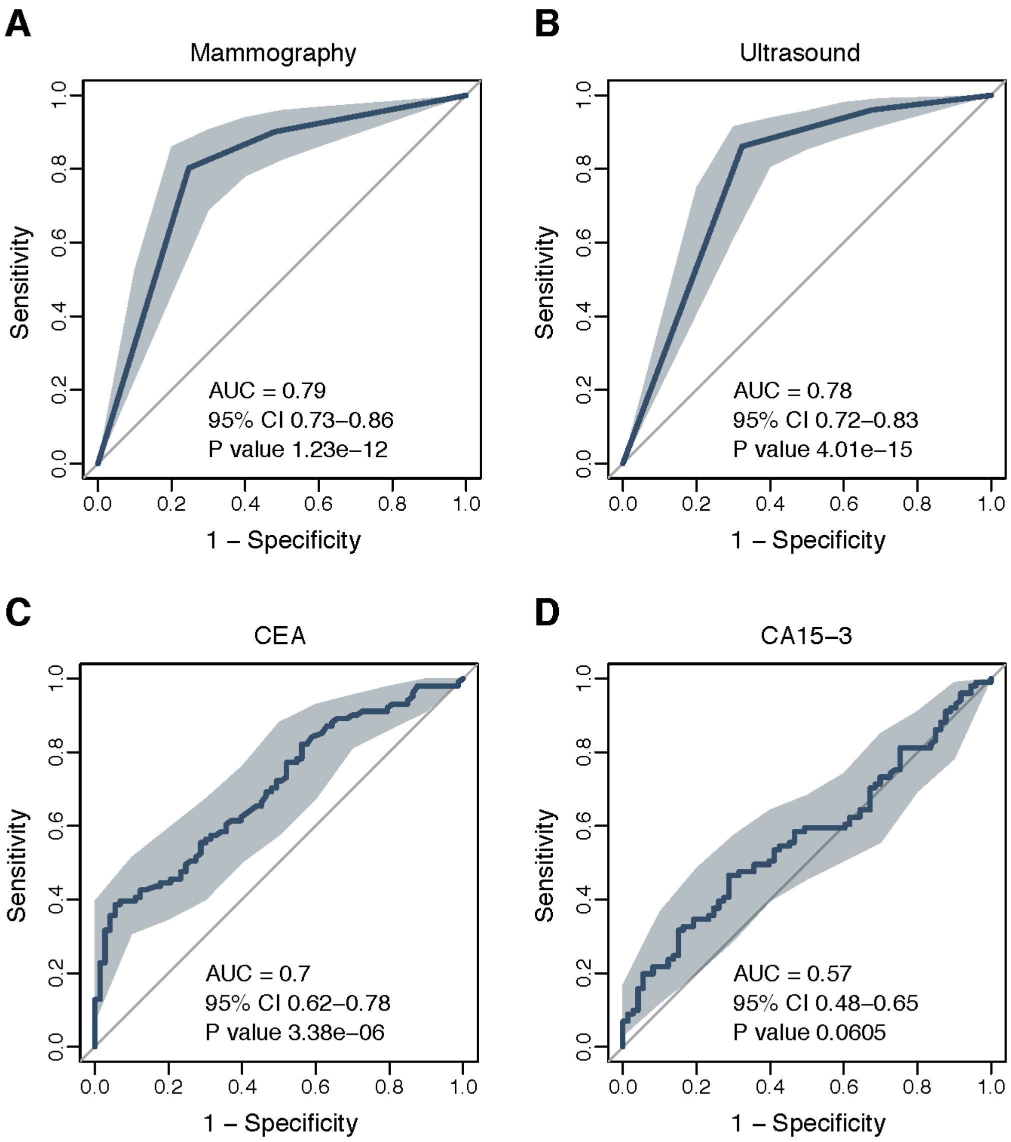

Fig. S5. Receiver operating characteristic (ROC) curves of the diagnostic prediction model using mammography (A), ultrasound (B), CEA (C), and CA15-3 (D). The area under the curve (AUC) of the BI-RADS findings (AUC=0.78-0.79 for mammography and ultrasound) or relevant tumor biomarkers (AUC=0.57-0.70 for CA15-3 and CEA). The 95% confidence interval (CI) was shown as the grey area.

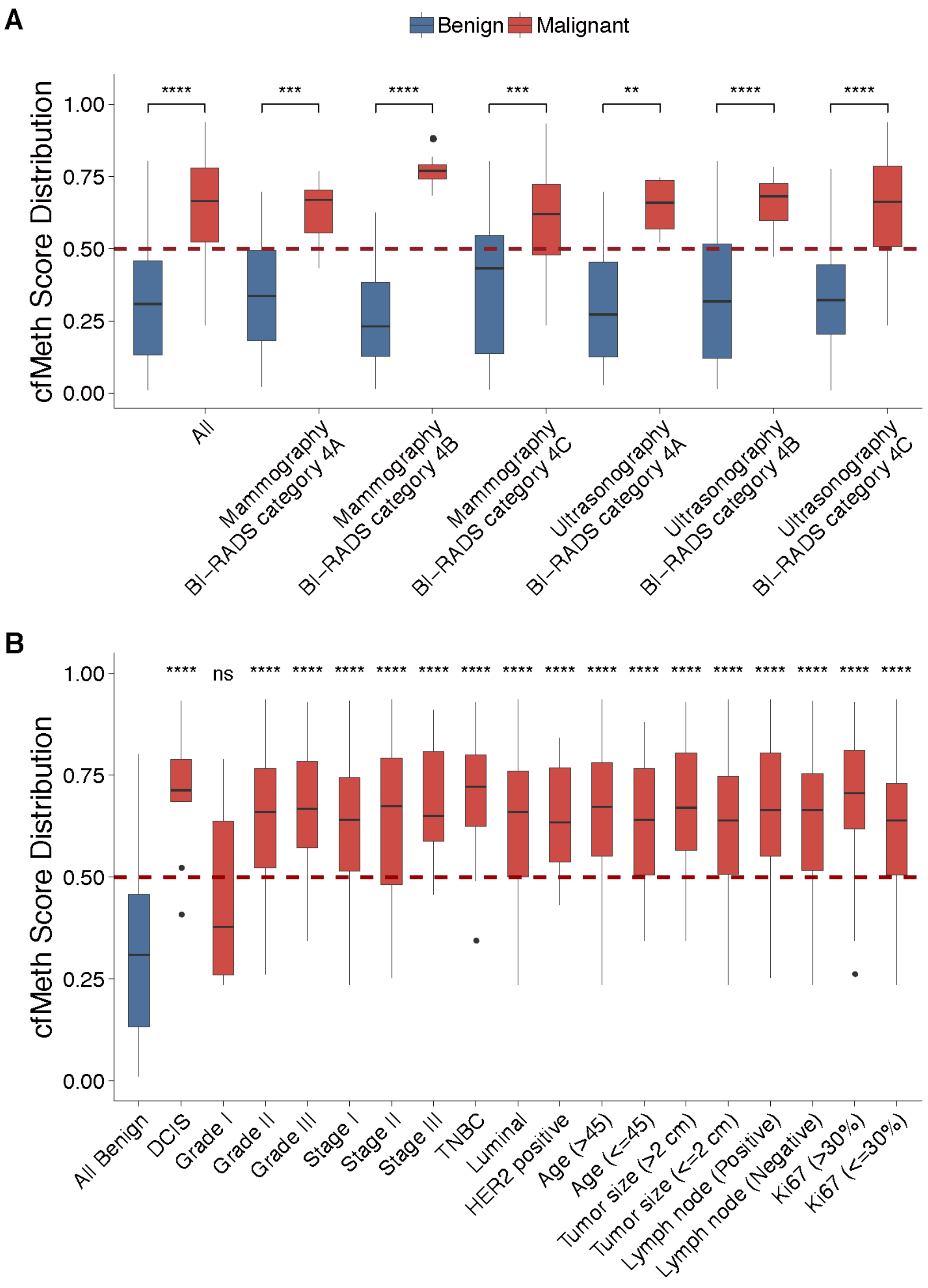

Fig. S6. Evaluating cfMeth score in categories of the Breast Imaging Reporting and Data System (BI-RADS) and with the clinical and pathologic characteristics. (A), Differences were obtained by comparing the cfMeth scores in patients of each subcategory of the BI-RADS

category 4. **(B)**, Differences were obtained by comparing the cfMeth scores in patients of each clinical characteristics with patients with breast benign lesions. ns: not significant; * $p \leq 0.05$; ** $p \leq 0.01$; *** $p \leq 0.001$; **** $p \leq 0.0001$. Abbreviation: DCIS, ductal carcinoma *in situ*; TNBC, triple-negative breast cancer.

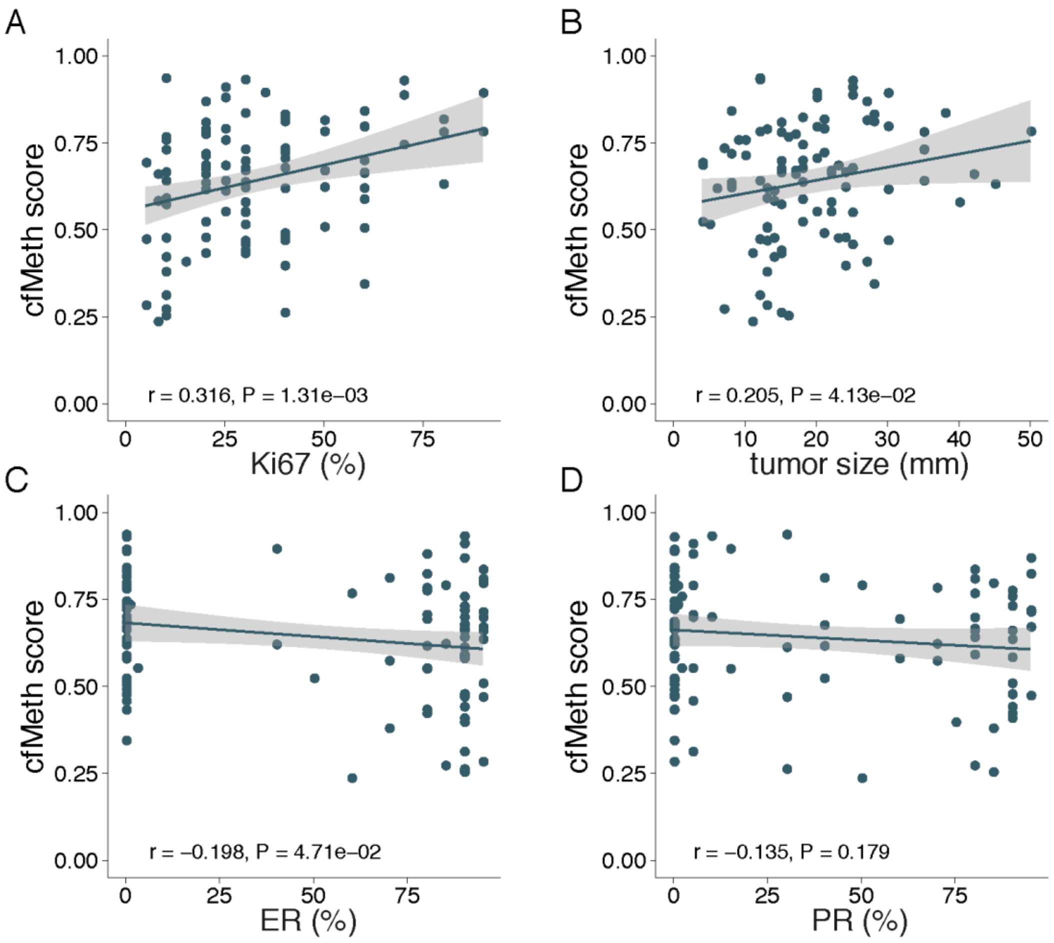

Fig. S7. The cfMeth score correlates ki-67, tumor size, estrogen receptor (ER) and progesterone receptor (PR). (**A**) and (**B**), Linear regression analysis was performed to assess the correlation and indicated that the cfMeth scores were collinear with proliferation fraction **(A)** and tumor size **(B)** (r=0.33 and 0.24, p=8.6×10^{-4} and 1.8×10^{-2}, respectively). **(C)** and **(D)**, However, the cfMeth scores were negatively correlated to ER status **(C)** (r=-0.22, p=3.0×10^{-2}) but not to PR status **(D)** (r=-0.16, p=0.10).

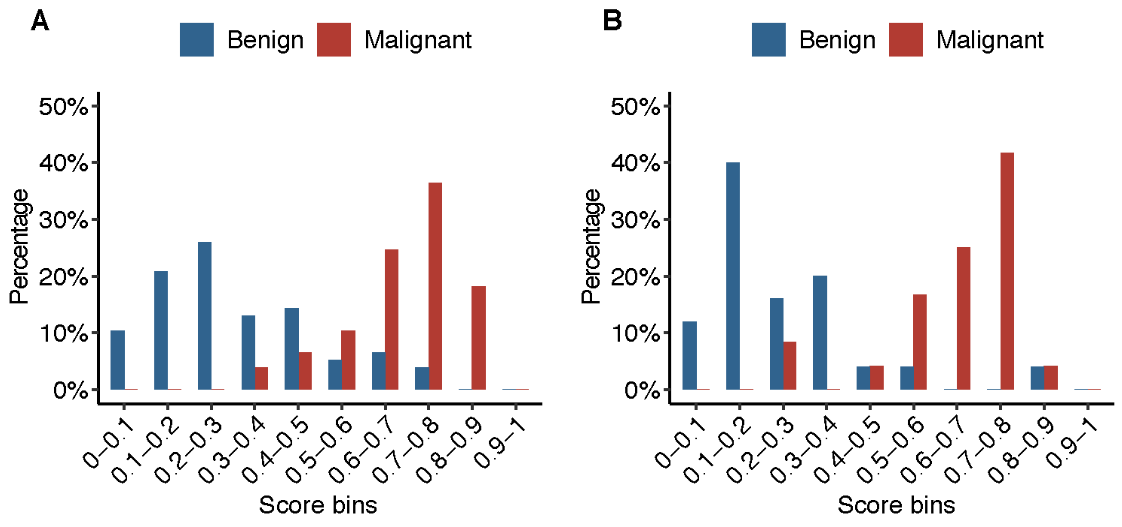

Fig. S8. The distributions of the combined scores in the patients with breast cancer (red) and patients with benign breast lesions (blue) in the discovery cohort (A) and the validation cohort (B). The combined scores were divided evenly into 10 bins and the bar height corresponds to the proportion of patients fall into each bin. The malignant and benign groups had statistically different distributions of the combined scores in both the discovery (p=1.4×10^{-30}) and validation cohorts (p=7.7×10^{-11}).

Table S1. Clinical characteristics of patients in the discovery and validation cohorts.

Characteristics	Tissue samples		Discovery cohort		Validation cohort	
	Malignant (N=9)	Benign (N=10)	Malignant (N=77)	Benign (N=77)	Malignant (N=24)	Benign (N=25)
Age, yr/o [a]	57.4±9.9	41.5±9.5	52.4±10.0	44.8±11.5	53.4±8.5	40.0±11.3
BI-RADS category in mammography [b]						
4a	11.1% (1/9)	30.0% (3/10)	5.2% (4/77)	29.9% (23/77)	16.7% (4/24)	68.0% (17/25)
4b	0% (0/9)	10.0% (1/10)	6.5% (5/77)	18.2% (14/77)	12.5% (3/24)	16.0% (4/25)
4c	44.4% (4/9)	30.0% (3/10)	62.3% (48/77)	22.1% (17/77)	70.8% (17/24)	8.0% (2/25)
4 without subcategory	44.4% (4/9)	30.0% (3/10)	26.0% (20/77)	29.9% (23/77)	0% (0/24)	8.0% (2/25)
BI-RADS category in ultrasound [b]						
4a	0% (0/9)	40.0% (4/10)	3.9% (3/77)	27.3% (21/77)	4.2% (1/24)	48.0% (12/25)
4b	11.1% (1/9)	40.0% (4/10)	7.8% (6/77)	35.1% (27/77)	16. 7% (4/24)	36.0% (9/25)
4c	88.9% (8/9)	20.0% (2/10)	88.3% (68/77)	37.7% (29/77)	79.2% (19/24)	16.0% (4/25)
4 without subcategory	0% (0/9)	0	0% (0/77)	0% (0/77)	0% (0/24)	0% (0/25)
Tumor size, No. (%)						
≤2 cm	77.8% (7/9)	-	52.0% (40/77)	-	83.3% (20/24)	-
2-5 cm	22.2% (2/9)	-	44.2% (34/77)	-	16.7% (4/24)	-
≥5 cm	0% (0/9)	-	1.3% (1/77)	-	0% (0/24)	-
Histology grade, No. (%)						
in situ	0% (0/9)	-	10.4% (8/77)	-	4.2% (1/24)	-
G1	11.1% (1/9)	-	5.2% (4/77)	-	8.3% (2/24)	-
G2	44.4% (4/9)	-	40.3% (31/77)	-	58.3% (14/24)	-
G3	44.4% (4/9)	-	44.2% (34/77)	-	25.0% (6/24)	-
Stage [c], No. (%)						
I	44.4% (4/9)	-	37.7% (29/77)	-	66.7% (16/24)	-
II	44.4% (4/9)	-	36.4% (28/77)	-	25. 0% (6/24)	-
III	11.1% (1/9)	-	26.0% (20/77)	-	8.3% (2/24)	-
Molecular subtype, No. (%)						
Luminal	77.8% (7/9)	-	66.2% (51/77)	-	70.8% (17/24)	-
HER2+	11.1% (1/9)	-	15.6% (12/77)	-	25.0% (6/24)	-
TNBC	11.1% (1/9)	-	18.2% (14/77)	-	4.2% (1/24)	-

Serum tumor biomarkers						
CA15-3 positive	0% (0/9)	0% (0/10)	6.5% (5/77)	0% (0/50)	0% (0/24)	0% (0/23)
CEA positive	22.2% (2/9)	0% (0/10)	9.1% (7/77)	0% (0/50)	0% (0/24)	0% (0/23)

[a] Data was shown as the mean ± standard deviation.

[b] Categories of the Breast Imaging Reporting and Data System.

[c] Clinical staging was determined according to the eighth edition of the classification for breast cancer of the American Joint Commission of Cancer.

Abbreviation: -, not available; TNBC, triple-negative breast cancer.

Table S2. Summary of patients' clinical information in discovery and validation cohorts.

Patient ID	Age	Gender	Mammo-graphy	Ultrasono-graphy	CA-153	CEA	Pathology	Tumor size	Histology grade	Lymph node status	AJCC stage	Molecular subtype
NCC001	73	Female	4	4C	17.49	10.85	Malignant	>2 cm	III	Positive	III	TNBC
NCC002	67	Female	4C	4C	6.69	6.45	Malignant	≤2 cm	I	Positive	II	Luminal
NCC003	33	Female	4C	4B	5.84	2.08	Benign	NA	NA	NA	NA	NA
NCC004	49	Female	4A	4B	11.40	1.77	Benign	NA	NA	NA	NA	NA
NCC005	59	Female	4A	4C	6.52	1.46	Malignant	≤2 cm	III	Negative	I	Luminal
NCC006	37	Female	4C	4B	7.07	1.25	Benign	NA	NA	NA	NA	NA
NCC007	51	Female	4C	4B	14.51	4.99	Malignant	≤2 cm	II	Positive	II	Luminal
NCC008	41	Female	4C	4C	9.32	1.55	Malignant	≤2 cm	III	Negative	I	Luminal
NCC009	45	Female	4A	4C	7.97	1.84	Benign	NA	NA	NA	NA	NA
NCC010	69	Female	4C	4C	52.01	2.11	Malignant	>2 cm	II	Positive	III	Luminal
NCC011	62	Female	4	4C	8.70	0.76	Malignant	≤2 cm	III	Negative	I	Luminal
NCC012	55	Female	4C	4C	29.47	1.23	Malignant	>2 cm	III	Negative	II	TNBC
NCC013	34	Female	4	4B	NA	NA	Benign	NA	NA	NA	NA	NA
NCC014	28	Female	4	4B	11.41	0.30	Benign	NA	NA	NA	NA	NA
NCC015	65	Female	4	4C	17.01	1.36	Malignant	≤2 cm	II	Positive	II	Luminal
NCC016	67	Female	4A	4C	10.14	1.37	Benign	NA	NA	NA	NA	NA
NCC017	56	Female	4C	4C	4.90	2.33	Malignant	>2 cm	III	Positive	II	HER2+
NCC018	57	Female	4	4C	9.46	1.96	Malignant	≤2 cm	II	Negative	I	Luminal
NCC019	53	Female	4	4C	26.81	4.74	Malignant	>2 cm	III	Negative	II	Luminal
NCC020	40	Female	4B	4C	10.54	0.50	Malignant	>2 cm	III	Positive	II	TNBC
NCC021	42	Female	4C	4C	7.22	2.29	Benign	NA	NA	NA	NA	NA
NCC022	53	Female	4C	4C	9.80	2.60	Malignant	≤2 cm	III	Negative	I	Luminal
NCC023	52	Female	4C	4C	15.75	2.07	Malignant	>2 cm	II	Positive	III	Luminal
NCC024	44	Female	4C	4C	4.81	2.81	Malignant	≤2 cm	III	Negative	I	Luminal
NCC025	52	Female	4C	4C	19.19	2.60	Malignant	>2 cm	II	Positive	II	Luminal
NCC026	50	Female	4C	4C	8.00	2.24	Malignant	≤2 cm	III	Negative	I	Luminal

Patient ID	Age	Gender	Mammo-graphy	Ultrasono-graphy	CA-153	CEA	Pathology	Tumor size	Histology grade	Lymph node status	AJCC stage	Molecular subtype
NCC027	72	Female	4C	4C	14.28	5.58	Malignant	>2 cm	III	Positive	II	HER2+
NCC028	44	Female	4	4B	6.25	0.58	Benign	NA	NA	NA	NA	NA
NCC029	53	Female	4C	4C	5.27	0.89	Malignant	≤2 cm	III	Negative	I	Luminal
NCC030	47	Female	4C	4C	6.00	0.20	Malignant	≤2 cm	I	Negative	I	Luminal
NCC031	61	Female	4	4C	6.83	0.51	Malignant	≤2 cm	II	Positive	III	Luminal
NCC032	46	Female	4C	4C	2.90	1.54	Benign	NA	NA	NA	NA	NA
NCC033	38	Female	4B	4B	6.23	0.46	Benign	NA	NA	NA	NA	NA
NCC034	65	Female	4C	4C	6.50	0.51	Benign	NA	NA	NA	NA	NA
NCC035	49	Female	4C	4C	12.25	1.61	Malignant	≤2 cm	II	Positive	II	Luminal
NCC036	59	Female	4	4C	10.16	0.89	Malignant	NA	in situ	Negative	I	Luminal
NCC037	27	Female	4	4A	NA	NA	Benign	NA	NA	NA	NA	NA
NCC038	38	Female	4	4B	NA	NA	Benign	NA	NA	NA	NA	NA
NCC039	41	Female	4	4C	20.02	2.94	Malignant	≤2 cm	III	Positive	II	Luminal
NCC040	46	Female	4C	4C	10.00	1.13	Malignant	≤2 cm	II	Negative	I	Luminal
NCC041	60	Female	4C	4C	10.87	2.25	Malignant	>2 cm	II	Negative	II	Luminal
NCC042	63	Female	4	4C	7.96	3.15	Malignant	≤2 cm	II	Negative	I	Luminal
NCC043	50	Female	4A	4C	11.75	0.59	Benign	NA	NA	NA	NA	NA
NCC044	36	Female	4B	4A	13.12	1.34	Malignant	≤2 cm	III	Positive	II	Luminal
NCC045	56	Female	4B	4B	2.55	1.67	Benign	NA	NA	NA	NA	NA
NCC046	63	Female	4C	4C	15.65	4.64	Malignant	>2 cm	II	Positive	II	HER2+
NCC047	56	Female	4A	4C	12.36	10.75	Malignant	≤2 cm	II	Negative	I	Luminal
NCC048	62	Female	4B	4C	9.07	0.37	Benign	NA	NA	NA	NA	NA
NCC049	58	Female	4C	4C	6.74	0.74	Malignant	≤2 cm	I	Negative	I	Luminal
NCC050	37	Female	4C	4C	6.80	0.93	Malignant	>2 cm	III	Negative	II	TNBC
NCC051	44	Female	4	4C	5.18	0.48	Malignant	≤2 cm	II	Negative	I	Luminal
NCC052	50	Female	4	4B	11.59	1.11	Malignant	≤2 cm	II	Positive	III	Luminal
NCC053	37	Female	4C	4C	11.34	1.45	Malignant	≤2 cm	II	Negative	I	Luminal
NCC054	65	Female	4C	4C	11.32	3.77	Malignant	>2 cm	II	Positive	III	Luminal

Patient ID	Age	Gender	Mammo-graphy	Ultrasono-graphy	CA-153	CEA	Pathology	Tumor size	Histology grade	Lymph node status	AJCC stage	Molecular subtype
NCC055	50	Female	4C	4C	7.11	3.11	Malignant	>2 cm	III	Positive	III	Luminal
NCC056	57	Female	4B	4B	13.29	3.65	Benign	NA	NA	NA	NA	NA
NCC057	55	Female	4	4C	10.17	1.21	Malignant	≤2 cm	I	Negative	I	Luminal
NCC058	30	Female	4	4C	13.49	0.93	Malignant	≤2 cm	III	Negative	I	Luminal
NCC059	50	Female	4	4C	6.25	1.03	Malignant	NA	in situ	Negative	I	Luminal
NCC060	40	Female	4	4C	5.37	1.02	Malignant	≤2 cm	II	Negative	I	HER2+
NCC061	32	Female	4B	4C	12.57	1.58	Malignant	≤2 cm	in situ	Positive	III	Luminal
NCC062	41	Female	4	4C	9.29	0.45	Benign	NA	NA	NA	NA	NA
NCC063	20	Female	4	4A	NA	NA	Benign	NA	NA	NA	NA	NA
NCC064	45	Female	4C	4C	5.53	1.16	Malignant	>2 cm	III	Positive	III	Luminal
NCC065	43	Female	4C	4C	6.52	2.13	Malignant	>2 cm	II	Positive	III	Luminal
NCC066	52	Female	4C	4B	7.48	0.73	Benign	NA	NA	NA	NA	NA
NCC067	57	Female	4C	4C	12.46	1.62	Malignant	>2 cm	II	Positive	III	Luminal
NCC068	45	Female	4A	4B	4.66	1.72	Benign	NA	NA	NA	NA	NA
NCC069	47	Female	4C	4A	4.86	0.48	Benign	NA	NA	NA	NA	NA
NCC070	68	Female	4C	4C	7.58	2.23	Malignant	≤2 cm	II	Negative	I	Luminal
NCC071	31	Female	4B	4C	7.33	2.04	Benign	NA	NA	NA	NA	NA
NCC072	49	Female	4B	4B	20.60	0.88	Benign	NA	NA	NA	NA	NA
NCC073	54	Female	4C	4B	8.92	0.54	Benign	NA	NA	NA	NA	NA
NCC074	29	Female	4	4B	9.91	1.22	Benign	NA	NA	NA	NA	NA
NCC075	53	Female	4B	4C	5.81	0.87	Benign	NA	NA	NA	NA	NA
NCC076	45	Female	4C	4C	5.67	1.36	Benign	NA	NA	NA	NA	NA
NCC077	52	Female	4C	4C	6.12	0.52	Malignant	>2 cm	III	Positive	II	Luminal
NCC078	46	Female	4B	4A	5.93	2.01	Benign	NA	NA	NA	NA	NA
NCC079	62	Female	4C	4C	7.61	2.00	Malignant	>2 cm	II	Positive	II	Luminal
NCC081	45	Female	4A	4C	7.41	1.56	Benign	NA	NA	NA	NA	NA
NCC082	51	Female	4C	4C	33.92	6.56	Malignant	>2 cm	III	Positive	III	HER2+
NCC083	42	Female	4C	4C	9.17	0.48	Malignant	≤2 cm	III	Positive	III	HER2+

Patient ID	Age	Gender	Mammo-graphy	Ultrasono-graphy	CA-153	CEA	Pathology	Tumor size	Histology grade	Lymph node status	AJCC stage	Molecular subtype
NCC080	57	Female	4C	4A	5.67	1.41	Benign	NA	NA	NA	NA	NA
NCC084	44	Female	4C	4C	NA	NA	Benign	NA	NA	NA	NA	NA
NCC085	46	Female	4A	4A	14.06	1.58	Benign	NA	NA	NA	NA	NA
NCC086	45	Female	4	4C	5.47	1.21	Benign	NA	NA	NA	NA	NA
NCC087	42	Female	4C	4C	11.22	0.20	Benign	NA	NA	NA	NA	NA
NCC088	45	Female	4C	4C	4.31	0.60	Malignant	>2 cm	II	Positive	III	Luminal
NCC089	69	Female	4C	4C	12.02	0.87	Malignant	>2 cm	III	Negative	II	HER2+
NCC090	47	Female	4B	4B	6.77	0.77	Benign	NA	NA	NA	NA	NA
NCC091	55	Female	4C	4C	16.50	1.26	Malignant	>2 cm	III	Negative	II	HER2+
NCC092	61	Female	4C	4C	20.55	3.24	Malignant	>2 cm	III	Positive	III	Luminal
NCC093	38	Female	4C	4C	15.45	6.59	Malignant	>2 cm	III	Positive	II	HER2+
NCC094	29	Female	4	4B	6.76	0.34	Benign	NA	NA	NA	NA	NA
NCC095	47	Female	4	4C	5.15	0.59	Malignant	≤2 cm	III	Negative	I	Luminal
NCC096	34	Female	4A	4A	7.30	0.65	Benign	NA	NA	NA	NA	NA
NCC097	58	Female	4	4C	44.69	4.92	Malignant	≤2 cm	II	Positive	III	HER2+
NCC099	45	Female	4C	4C	6.06	2.20	Malignant	≤2 cm	III	Positive	III	TNBC
NCC100	63	Female	4A	4B	10.99	1.12	Benign	NA	NA	NA	NA	NA
NCC102	48	Female	4C	4C	8.76	2.54	Malignant	≤2 cm	II	Positive	II	Luminal
NCC105	42	Female	4	4A	7.73	0.79	Benign	NA	NA	NA	NA	NA
NCC106	22	Female	4	4A	13.57	1.19	Benign	NA	NA	NA	NA	NA
NCC107	34	Female	4	4A	NA	NA	Benign	NA	NA	NA	NA	NA
NCC108	56	Female	4A	4A	NA	NA	Benign	NA	NA	NA	NA	NA
NCC109	28	Female	4	4B	NA	NA	Benign	NA	NA	NA	NA	NA
NCC110	41	Female	4A	4A	NA	NA	Benign	NA	NA	NA	NA	NA
NCC113	47	Female	4A	4B	NA	NA	Benign	NA	NA	NA	NA	NA
NCC114	39	Female	4	4B	NA	NA	Benign	NA	NA	NA	NA	NA
NCC115	50	Female	4A	4A	NA	NA	Benign	NA	NA	NA	NA	NA
NCC116	63	Female	4A	4A	NA	NA	Benign	NA	NA	NA	NA	NA

Patient ID	Age	Gender	Mammo-graphy	Ultrasono-graphy	CA-153	CEA	Pathology	Tumor size	Histology grade	Lymph node status	AJCC stage	Molecular subtype
NCC111	46	Female	4A	4A	NA	NA	Benign	NA	NA	NA	NA	NA
NCC112	26	Female	4	4A	NA	NA	Benign	NA	NA	NA	NA	NA
NCC117	55	Female	4A	4A	NA	NA	Benign	NA	NA	NA	NA	NA
NCC118	58	Female	4A	4A	NA	NA	Benign	NA	NA	NA	NA	NA
NCC119	51	Female	4A	4A	NA	NA	Benign	NA	NA	NA	NA	NA
NCC120	35	Female	4	4B	NA	NA	Benign	NA	NA	NA	NA	NA
NCC121	24	Female	4	4A	NA	NA	Benign	NA	NA	NA	NA	NA
NCC122	67	Female	4B	4B	NA	NA	Benign	NA	NA	NA	NA	NA
NCC123	63	Female	4A	4C	NA	NA	Benign	NA	NA	NA	NA	NA
NCC124	62	Female	4B	4B	NA	NA	Benign	NA	NA	NA	NA	NA
NCC125	43	Female	4A	4B	NA	NA	Benign	NA	NA	NA	NA	NA
NCC127	64	Female	4B	4C	NA	NA	Benign	NA	NA	NA	NA	NA
NCC128	37	Female	4	4C	NA	NA	Benign	NA	NA	NA	NA	NA
NCC129	34	Female	4	4B	NA	NA	Benign	NA	NA	NA	NA	NA
NCC130	48	Female	4C	4C	7.82	1.84	Benign	NA	NA	NA	NA	NA
NCC131	49	Female	4C	4C	6.39	1.17	Malignant	≤2 cm	in situ	Negative	I	Luminal
NCC132	36	Female	4	4C	9.18	1.09	Benign	NA	NA	NA	NA	NA
NCC133	53	Female	4C	4C	7.65	3.05	Malignant	>2 cm	III	Negative	II	TNBC
NCC134	40	Female	4B	4C	10.90	1.44	Benign	NA	NA	NA	NA	NA
NCC136	51	Female	4	4C	6.15	1.09	Malignant	>2 cm	II	Positive	III	Luminal
NCC137	46	Female	4	4C	6.33	1.26	Benign	NA	NA	NA	NA	NA
NCC138	52	Female	4	4C	19.54	0.74	Benign	NA	NA	NA	NA	NA
NCC139	39	Female	4A	4C	13.43	0.88	Malignant	>2 cm	II	Negative	II	TNBC
NCC145	44	Female	4C	4C	9.83	1.43	Malignant	≤2 cm	II	Negative	I	TNBC
NCC146	43	Female	4C	4C	7.23	1.14	Benign	NA	NA	NA	NA	NA
NCC147	60	Female	4C	4C	12.10	1.02	Malignant	≤2 cm	III	Negative	I	TNBC
NCC148	37	Female	4A	4C	4.42	1.80	Benign	NA	NA	NA	NA	NA
NCC149	69	Female	4	4C	7.57	2.54	Malignant	≤2 cm	III	Negative	I	TNBC

Patient ID	Age	Gender	Mammo-graphy	Ultrasono-graphy	CA-153	CEA	Pathology	Tumor size	Histology grade	Lymph node status	AJCC stage	Molecular subtype
NCC140	52	Female	4B	4A	8.04	3.43	Malignant	≤2 cm	II	Negative	I	TNBC
NCC141	32	Female	4B	4B	8.24	1.16	Malignant	>2 cm	III	Negative	II	TNBC
NCC143	52	Female	4C	4C	5.36	1.84	Malignant	>2 cm	in situ	Negative	II	Luminal
NCC150	47	Female	4A	4C	8.31	0.99	Benign	NA	NA	NA	NA	NA
NCC152	62	Female	4A	4B	12.52	2.43	Malignant	>2 cm	in situ	Negative	II	Luminal
NCC153	46	Female	4C	4A	12.94	0.96	Benign	NA	NA	NA	NA	NA
NCC154	65	Female	4B	4C	6.43	1.95	Benign	NA	NA	NA	NA	NA
NCC155	43	Female	4A	4B	14.63	1.00	Benign	NA	NA	NA	NA	NA
NCC156	63	Female	4C	4C	16.22	4.34	Malignant	>2 cm	II	Positive	III	Luminal
NCC157	47	Female	4C	4A	16.88	1.40	Malignant	≤2 cm	in situ	Negative	I	HER2+
NCC160	66	Female	4	4B	7.42	3.12	Malignant	≤2 cm	in situ	Negative	I	Luminal
NCC161	54	Female	4C	4C	15.83	1.54	Malignant	>2 cm	II	Positive	III	HER2+
NCC162	54	Female	4C	4C	18.74	1.75	Benign	NA	NA	NA	NA	NA
NCC164	27	Female	4C	4C	8.93	2.01	Benign	NA	NA	NA	NA	NA
NCC167	48	Female	4C	4B	20.64	2.63	Malignant	>2 cm	III	Negative	II	TNBC
NCC168	56	Female	4C	4C	15.49	5.99	Malignant	>2 cm	III	Positive	II	TNBC
HAR005	54	Female	4C	4C	6.55	3.54	Malignant	>2 cm	III	Positive	II	Luminal
HAR006	45	Female	4C	4C	6.35	0.79	Malignant	≤2 cm	II	Negative	I	Luminal
HAR015	54	Female	4C	4B	2.18	1.22	Malignant	≤2 cm	II	Negative	I	Luminal
HAR017	50	Female	4A	4B	7.33	0.56	Benign	NA	NA	NA	NA	NA
HAR018	48	Female	4A	4A	NA	NA	Benign	NA	NA	NA	NA	NA
HAR019	22	Female	4A	4C	6.34	0.78	Benign	NA	NA	NA	NA	NA
HAR028	42	Female	4A	4B	3.76	1.99	Benign	NA	NA	NA	NA	NA
HAR029	38	Female	4A	4B	2.83	0.89	Benign	NA	NA	NA	NA	NA
HAR030	38	Female	4B	4A	8.34	0.75	Benign	NA	NA	NA	NA	NA
HAR031	59	Female	4A	4B	13.58	2.68	Benign	NA	NA	NA	NA	NA
HAR032	39	Female	4A	4A	3.86	1.1	Benign	NA	NA	NA	NA	NA
HAR033	29	Female	4A	4A	7.39	1.64	Benign	NA	NA	NA	NA	NA

Patient ID	Age	Gender	Mammo-graphy	Ultrasono-graphy	CA-153	CEA	Pathology	Tumor size	Histology grade	Lymph node status	AJCC stage	Molecular subtype
HAR020	25	Female	4A	4A	7.33	0.34	Benign	NA	NA	NA	NA	NA
HAR023	24	Female	4	4A	NA	NA	Benign	NA	NA	NA	NA	NA
HAR025	66	Female	4C	4C	6.7	3.1	Malignant	≤2 cm	III	Positive	III	Luminal
HAR026	61	Female	4C	4C	22.15	1.66	Malignant	>2 cm	III	Positive	II	TNBC
HAR034	70	Female	4B	4C	6.15	1.11	Malignant	≤2 cm	II	Negative	I	Luminal
HAR038	56	Female	4C	4C	15.78	2.15	Benign	NA	NA	NA	NA	NA
HAR039	41	Female	4A	4C	6.47	1.28	Malignant	≤2 cm	II	Negative	I	Luminal
HAR040	44	Female	4A	4B	8.62	0.45	Benign	NA	NA	NA	NA	NA
HAR042	30	Female	4A	4A	7.44	0.66	Benign	NA	NA	NA	NA	NA
HAR043	45	Female	4C	4C	8.03	2.13	Malignant	≤2 cm	II	Positive	II	Luminal
HAR047	50	Female	4C	4C	4.51	1.79	Malignant	≤2 cm	II	Negative	I	Luminal
HAR048	33	Female	4A	4A	3.54	0.88	Benign	NA	NA	NA	NA	NA
HAR049	58	Female	4A	4B	10.22	1.35	Benign	NA	NA	NA	NA	NA
HAR050	46	Female	4A	4B	5.13	0.81	Malignant	≤2 cm	I	Negative	I	Luminal
HAR051	44	Female	4C	4C	7.04	2.37	Malignant	>2 cm	III	Positive	III	HER2+
HAR052	51	Female	4C	4C	3.37	1.74	Malignant	≤2 cm	III	Negative	I	HER2+
HAR054	60	Female	4C	4C	4.29	1.2	Malignant	≤2 cm	III	Negative	I	HER2+
HAR055	48	Female	4B	4C	3.74	0.92	Malignant	≤2 cm	II	Negative	I	HER2+
HAR056	56	Female	4C	4C	3.62	1.15	Malignant	≤2 cm	in situ	Negative	I	HER2+
HAR057	34	Female	4B	4C	5.95	0.43	Benign	NA	NA	NA	NA	NA
HAR058	66	Female	4C	4C	7.04	1.65	Malignant	≤2 cm	II	Positive	II	Luminal
HAR066	46	Female	4A	4B	10.63	0.76	Benign	NA	NA	NA	NA	NA
HAR069	46	Female	4B	4B	8.2	0.62	Benign	NA	NA	NA	NA	NA
HAR070	50	Female	4B	4C	9.7	0.92	Malignant	>2 cm	II	Negative	II	HER2+
HAR071	48	Female	4C	4A	5.16	1.2	Benign	NA	NA	NA	NA	NA
HAR072	22	Female	4	4A	6.85	0.86	Benign	NA	NA	NA	NA	NA
HAR073	47	Female	4A	4C	5.32	1.25	Benign	NA	NA	NA	NA	NA
HAR074	41	Female	4C	4C	6.18	0.97	Malignant	≤2 cm	II	Positive	II	Luminal

Patient ID	Age	Gender	Mammo-graphy	Ultrasono-graphy	CA-153	CEA	Pathology	Tumor size	Histology grade	Lymph node status	AJCC stage	Molecular subtype
HAR060	52	Female	4C	4B	6.27	1.96	Malignant	≤2 cm	II	Negative	I	Luminal
HAR061	44	Female	4B	4A	7.64	1.16	Benign	NA	NA	NA	NA	NA
HAR063	27	Female	4A	4A	15.21	0.9	Benign	NA	NA	NA	NA	NA
HAR064	48	Female	4C	4C	5.47	0.28	Malignant	≤2 cm	II	Negative	I	Luminal
HAR065	66	Female	4C	4C	4.42	0.87	Malignant	≤2 cm	II	Negative	I	Luminal
HAR075	54	Female	4A	4B	5.39	1.46	Malignant	≤2 cm	I	Negative	I	Luminal
HAR076	50	Female	4A	4B	6.91	0.69	Benign	NA	NA	NA	NA	NA
HAR078	50	Female	4A	4A	9.37	1.05	Malignant	≤2 cm	NA	Negative	I	Luminal
HAR079	64	Female	4C	4C	10.28	2.4	Malignant	≤2 cm	II	Negative	I	Luminal

Table S3. Summary of samples and WGBS information.

Patient ID	Source of DNA	Sample type	Sample volume	cfDNA amount	Depth	Mapping efficiency	Conversion efficiency
NCC001-T	Genomic DNA	Tissue	NA	12720.0	14.6	83.6	99.6
NCC002-T	Genomic DNA	Tissue	NA	6380.0	13.7	84.7	99.6
NCC003-T	Genomic DNA	Tissue	NA	6780.0	13.0	82.9	98.7
NCC004-T	Genomic DNA	Tissue	NA	1340.0	13.8	85.6	99.6
NCC005-T	Genomic DNA	Tissue	NA	7220.0	14.5	86.1	99.6
NCC006-T	Genomic DNA	Tissue	NA	9260.0	13.1	83.7	99.5
NCC007-T	Genomic DNA	Tissue	NA	16320.0	9.5	82.2	99.6
NCC009-T	Genomic DNA	Tissue	NA	7040.0	12.6	85.1	99.4
NCC015-T	Genomic DNA	Tissue	NA	1480.0	10.2	82.3	99.6
NCC018-T	Genomic DNA	Tissue	NA	2460.0	12.2	82.6	99.6
NCC022-T	Genomic DNA	Tissue	NA	2180.0	16.6	82.8	99.5
NCC060-T	Genomic DNA	Tissue	NA	3420.0	13.1	82.2	99.6
NCC077-T	Genomic DNA	Tissue	NA	1420.0	13.5	82.1	99.6
NCC078-T	Genomic DNA	Tissue	NA	8520.0	10.4	82.7	99.5
NCC080-T	Genomic DNA	Tissue	NA	6200.0	10.9	82.4	99.5
NCC081-T	Genomic DNA	Tissue	NA	4100.0	9.2	82.5	99.6
NCC105-T	Genomic DNA	Tissue	NA	6700.0	9.8	83.9	99.5
NCC106-T	Genomic DNA	Tissue	NA	5460.0	10.2	83.3	99.5
NCC114-T	Genomic DNA	Tissue	NA	1620.0	11.6	81.9	99.5
NCC001	cfDNA	Whole blood	NA	42.8	10.8	87.2	99.6
NCC002	cfDNA	Whole blood	NA	64.4	13.8	86.1	99.6
NCC003	cfDNA	Whole blood	NA	46.8	10.5	86.1	99.6
NCC004	cfDNA	Whole blood	NA	59.9	14.7	86.1	99.6
NCC005	cfDNA	Whole blood	NA	42.6	12.2	86.8	99.6
NCC006	cfDNA	Whole blood	NA	48.7	12.3	86.1	99.6
NCC007	cfDNA	Whole blood	NA	48.2	7.2	73.9	99.2
NCC008	cfDNA	Whole blood	7.0	43.7	6.7	75.6	99.0
NCC009	cfDNA	Whole blood	NA	25.7	13.0	85.5	99.5
NCC010	cfDNA	Whole blood	7.8	40.0	12.8	85.9	99.6
NCC011	cfDNA	Whole blood	7.0	45.4	13.2	86.2	99.4
NCC012	cfDNA	Whole blood	5.0	79.0	5.4	67.9	99.2
NCC013	cfDNA	Whole blood	7.0	25.1	15.3	84.5	99.6
NCC014	cfDNA	Whole blood	4.0	21.2	15.1	84.2	99.7
NCC015	cfDNA	Whole blood	7.5	80.1	13.4	85.0	99.6
NCC016	cfDNA	Whole blood	7.0	65.5	13.5	85.6	99.7
NCC017	cfDNA	Whole blood	7.0	42.0	13.4	86.4	99.6
NCC018	cfDNA	Whole blood	7.2	43.4	12.3	86.3	99.6
NCC019	cfDNA	Whole blood	6.0	75.0	7.9	73.3	99.2
NCC020	cfDNA	Whole blood	6.2	41.7	10.2	85.2	99.6
NCC021	cfDNA	Whole blood	6.2	34.2	11.9	85.0	99.6

Patient ID	Source of DNA	Sample type	Sample volume	cfDNA amount	Depth	Mapping efficiency	Conversion efficiency
NCC022	cfDNA	Whole blood	6.5	73.9	11.8	87.4	99.4
NCC023	cfDNA	Whole blood	6.5	57.1	12.5	86.6	99.6
NCC024	cfDNA	Whole blood	6.5	55.4	15.4	86.7	99.3
NCC025	cfDNA	Whole blood	6.5	49.6	11.1	85.7	99.6
NCC026	cfDNA	Whole blood	6.5	51.5	11.3	85.8	99.6
NCC027	cfDNA	Whole blood	6.5	38.6	10.9	78.8	99.6
NCC028	cfDNA	Whole blood	6.5	57.7	11.2	78.6	99.6
NCC029	cfDNA	Whole blood	6.5	110.9	5.8	71.4	99.2
NCC030	cfDNA	Whole blood	6.5	71.7	7.0	71.2	99.3
NCC031	cfDNA	Whole blood	6.0	116.5	10.4	85.6	99.6
NCC032	cfDNA	Whole blood	5.5	62.7	10.5	78.4	99.6
NCC033	cfDNA	Whole blood	5.4	193.8	13.9	86.4	99.6
NCC034	cfDNA	Whole blood	6.0	62.2	14.4	86.7	99.7
NCC035	cfDNA	Whole blood	6.4	79.5	10.9	86.7	99.7
NCC036	cfDNA	Whole blood	6.3	85.1	4.5	64.1	99.4
NCC037	cfDNA	Whole blood	7.8	68.9	13.4	83.9	99.6
NCC038	cfDNA	Whole blood	7.8	64.4	11.9	84.9	99.6
NCC039	cfDNA	Whole blood	6.8	47.0	12.9	83.3	99.6
NCC040	cfDNA	Whole blood	6.8	53.2	10.6	78.5	99.6
NCC041	cfDNA	Whole blood	6.5	25.1	10.1	78.7	97.5
NCC042	cfDNA	Whole blood	7.0	68.3	10.5	78.9	99.6
NCC043	cfDNA	Whole blood	6.5	51.0	9.1	80.4	99.6
NCC044	cfDNA	Whole blood	6.5	31.1	11.6	85.0	99.6
NCC045	cfDNA	Whole blood	7.0	26.9	12.1	85.5	99.6
NCC046	cfDNA	Whole blood	6.5	72.2	11.5	84.8	99.6
NCC047	cfDNA	Whole blood	7.0	110.9	11.1	84.5	99.6
NCC048	cfDNA	Whole blood	6.8	124.3	14.2	84.6	99.6
NCC049	cfDNA	Whole blood	6.5	111.4	10.7	85.0	99.6
NCC050	cfDNA	Whole blood	6.8	62.7	7.5	74.5	99.2
NCC051	cfDNA	Whole blood	6.5	114.8	13.1	85.2	99.6
NCC052	cfDNA	Whole blood	6.5	108.6	11.8	85.6	99.6
NCC053	cfDNA	Whole blood	6.8	34.4	13.0	85.1	98.9
NCC054	cfDNA	Whole blood	6.8	69.4	11.6	86.3	99.6
NCC055	cfDNA	Whole blood	6.8	51.5	11.1	85.9	99.6
NCC056	cfDNA	Whole blood	3.0	52.4	7.1	73.3	99.0
NCC057	cfDNA	Whole blood	6.8	47.0	14.8	86.3	99.6
NCC058	cfDNA	Whole blood	6.5	61.6	11.7	84.1	99.6
NCC059	cfDNA	Whole blood	6.8	65.5	13.8	86.1	99.4
NCC060	cfDNA	Whole blood	7.8	51.5	6.0	74.9	99.6
NCC061	cfDNA	Whole blood	7.5	67.2	10.3	78.0	99.6
NCC062	cfDNA	Whole blood	7.5	58.8	11.0	84.8	99.6
NCC063	cfDNA	Whole blood	6.8	44.2	6.9	73.4	98.8

Patient ID	Source of DNA	Sample type	Sample volume	cfDNA amount	Depth	Mapping efficiency	Conversion efficiency
NCC064	cfDNA	Whole blood	6.5	48.4	9.1	84.2	99.6
NCC065	cfDNA	Whole blood	6.5	80.1	14.0	83.8	99.6
NCC066	cfDNA	Whole blood	6.5	59.4	14.7	83.4	99.6
NCC067	cfDNA	Whole blood	7.0	44.8	4.0	78.3	99.6
NCC068	cfDNA	Whole blood	6.8	66.6	8.5	83.2	99.6
NCC069	cfDNA	Whole blood	6.8	93.5	10.2	84.1	99.7
NCC070	cfDNA	Whole blood	7.0	63.8	11.7	83.2	99.6
NCC071	cfDNA	Whole blood	6.8	44.2	10.4	83.5	99.6
NCC072	cfDNA	Whole blood	7.0	90.7	10.3	84.8	99.6
NCC073	cfDNA	Whole blood	6.0	44.0	11.5	84.2	99.6
NCC074	cfDNA	Whole blood	7.0	51.5	10.5	82.8	99.6
NCC075	cfDNA	Whole blood	7.0	70.0	16.1	84.5	99.6
NCC076	cfDNA	Whole blood	8.0	104.2	8.8	83.9	99.6
NCC077	cfDNA	Whole blood	7.0	84.6	10.5	84.0	99.6
NCC078	cfDNA	Whole blood	6.5	46.8	11.1	83.7	99.6
NCC079	cfDNA	Whole blood	6.8	42.8	6.8	76.8	99.6
NCC080	cfDNA	Whole blood	7.0	71.7	11.2	83.1	99.6
NCC081	cfDNA	Whole blood	6.8	66.6	9.1	82.5	99.6
NCC082	cfDNA	Whole blood	6.8	107.5	10.5	84.7	99.7
NCC083	cfDNA	Whole blood	7.0	96.3	11.1	85.0	99.6
NCC084	cfDNA	Whole blood	7.3	26.7	16.8	84.6	99.6
NCC085	cfDNA	Whole blood	7.0	52.1	9.1	82.3	99.6
NCC086	cfDNA	Whole blood	7.0	66.6	12.0	84.1	99.6
NCC087	cfDNA	Whole blood	7.0	80.1	10.9	84.4	99.6
NCC088	cfDNA	Whole blood	8.0	85.7	14.4	83.7	99.4
NCC089	cfDNA	Whole blood	8.5	68.9	9.3	84.3	99.6
NCC090	cfDNA	Whole blood	7.5	56.0	10.0	82.6	99.6
NCC091	cfDNA	Whole blood	7.0	49.6	11.5	84.2	99.6
NCC092	cfDNA	Whole blood	7.0	72.8	11.6	84.8	99.7
NCC093	cfDNA	Whole blood	7.0	53.5	15.5	83.4	99.6
NCC094	cfDNA	Whole blood	7.0	51.8	12.8	84.7	99.6
NCC095	cfDNA	Whole blood	7.0	40.3	12.3	85.2	99.6
NCC096	cfDNA	Whole blood	8.0	68.3	10.8	84.1	99.7
NCC097	cfDNA	Whole blood	8.0	74.5	10.8	84.5	99.6
NCC099	cfDNA	Whole blood	8.0	38.4	6.6	75.8	99.6
NCC100	cfDNA	Whole blood	7.5	54.3	9.4	83.5	99.6
NCC102	cfDNA	Whole blood	7.0	52.1	9.0	85.2	99.6
NCC105	cfDNA	Whole blood	7.5	33.9	9.1	83.0	99.6
NCC106	cfDNA	Whole blood	7.5	28.8	7.8	82.1	99.6
NCC107	cfDNA	Whole blood	7.0	79.0	10.4	83.8	99.6
NCC108	cfDNA	Whole blood	6.5	82.3	21.6	82.1	99.6
NCC109	cfDNA	Whole blood	6.0	70.6	10.2	83.1	99.6

Patient ID	Source of DNA	Sample type	Sample volume	cfDNA amount	Depth	Mapping efficiency	Conversion efficiency
NCC110	cfDNA	Whole blood	3.3	80.1	9.1	80.7	99.6
NCC111	cfDNA	Whole blood	7.0	61.6	9.4	83.9	99.6
NCC112	cfDNA	Whole blood	7.0	79.5	9.8	83.1	99.6
NCC113	cfDNA	Whole blood	7.0	92.4	10.4	84.5	99.6
NCC114	cfDNA	Whole blood	7.0	46.8	11.9	83.8	99.7
NCC115	cfDNA	Whole blood	6.8	93.0	12.1	83.3	99.6
NCC116	cfDNA	Whole blood	7.5	52.9	9.7	84.6	99.6
NCC117	cfDNA	Whole blood	7.0	76.7	10.6	83.8	99.6
NCC118	cfDNA	Whole blood	7.2	43.1	7.6	75.2	99.5
NCC119	cfDNA	Whole blood	8.0	41.2	15.5	81.2	99.6
NCC120	cfDNA	Whole blood	8.0	43.4	12.6	85.4	99.6
NCC121	cfDNA	Whole blood	7.0	43.7	11.5	83.5	99.6
NCC122	cfDNA	Whole blood	7.5	90.7	14.7	83.8	99.6
NCC123	cfDNA	Whole blood	7.5	59.4	11.2	83.6	99.6
NCC124	cfDNA	Whole blood	8.5	110.3	9.8	82.2	99.6
NCC125	cfDNA	Whole blood	8.5	47.9	9.7	82.0	99.6
NCC127	cfDNA	Whole blood	7.0	68.3	12.8	84.3	99.6
NCC128	cfDNA	Whole blood	9.0	67.8	9.9	85.5	99.7
NCC129	cfDNA	Whole blood	8.0	52.1	10.4	84.1	99.6
NCC130	cfDNA	Whole blood	NA	51.6	12.5	73.0	99.6
NCC131	cfDNA	Whole blood	NA	40.0	10.4	86.8	99.2
NCC132	cfDNA	Whole blood	NA	57.1	10.0	81.2	99.6
NCC133	cfDNA	Whole blood	NA	44.0	13.4	87.3	98.9
NCC134	cfDNA	Whole blood	NA	14.1	14.7	84.2	99.7
NCC136	cfDNA	Whole blood	NA	38.8	12.2	85.5	99.6
NCC137	cfDNA	Whole blood	NA	30.9	14.8	85.4	99.6
NCC138	cfDNA	Whole blood	NA	18.0	15.0	85.6	99.7
NCC139	cfDNA	Whole blood	NA	36.3	7.6	75.6	99.3
NCC140	cfDNA	Whole blood	NA	36.8	11.8	85.3	99.4
NCC141	cfDNA	Whole blood	NA	56.3	8.8	77.4	99.5
NCC143	cfDNA	Whole blood	NA	40.8	6.8	73.3	99.2
NCC145	cfDNA	Whole blood	NA	38.5	11.0	83.9	99.4
NCC146	cfDNA	Whole blood	NA	32.4	10.5	81.9	99.6
NCC147	cfDNA	Whole blood	NA	33.9	8.6	76.2	97.8
NCC148	cfDNA	Whole blood	NA	46.4	13.2	85.5	99.6
NCC149	cfDNA	Whole blood	NA	32.5	8.0	73.9	99.2
NCC150	cfDNA	Whole blood	NA	31.4	13.0	85.1	99.6
NCC152	cfDNA	Whole blood	NA	40.3	7.8	74.5	99.6
NCC153	cfDNA	Whole blood	NA	12.4	11.1	72.0	99.5
NCC154	cfDNA	Whole blood	NA	11.0	11.4	81.1	99.6
NCC155	cfDNA	Whole blood	NA	11.6	11.8	72.3	99.5
NCC156	cfDNA	Whole blood	NA	19.4	13.4	86.6	99.4

Patient ID	Source of DNA	Sample type	Sample volume	cfDNA amount	Depth	Mapping efficiency	Conversion efficiency
NCC157	cfDNA	Whole blood	NA	19.0	12.2	84.5	99.6
NCC160	cfDNA	Whole blood	NA	19.8	10.6	87.1	99.1
NCC161	cfDNA	Whole blood	NA	12.3	13.8	85.5	99.6
NCC162	cfDNA	Whole blood	NA	34.2	10.8	71.5	99.6
NCC164	cfDNA	Whole blood	NA	25.3	11.0	70.3	99.6
NCC167	cfDNA	Whole blood	NA	44.0	12.5	86.5	99.5
NCC168	cfDNA	Whole blood	NA	58.4	13.9	85.5	99.7
HAR005	cfDNA	Whole blood	8.0	56.6	11.7	84.6	99.7
HAR006	cfDNA	Whole blood	8.0	56.0	12.3	84.4	99.7
HAR015	cfDNA	Plasma	3.5	71.1	11.9	80.2	99.6
HAR017	cfDNA	Plasma	3.5	61.6	11.2	83.9	99.6
HAR018	cfDNA	Plasma	4.0	73.9	9.2	84.1	99.6
HAR019	cfDNA	Plasma	4.0	35.3	9.6	83.4	99.6
HAR020	cfDNA	Plasma	3.0	62.2	10.8	83.2	99.6
HAR023	cfDNA	Plasma	4.0	34.4	10.3	83.6	99.6
HAR025	cfDNA	Plasma	3.0	89.6	9.4	84.0	99.6
HAR026	cfDNA	Plasma	3.0	63.8	10.3	83.5	99.6
HAR028	cfDNA	Plasma	3.0	50.1	10.9	84.0	99.6
HAR029	cfDNA	Plasma	3.0	70.6	10.5	83.2	99.6
HAR030	cfDNA	Plasma	3.0	70.6	10.3	84.3	99.6
HAR031	cfDNA	Plasma	2.0	58.8	9.3	85.1	99.6
HAR032	cfDNA	Plasma	3.0	35.3	10.1	85.6	99.6
HAR033	cfDNA	Plasma	3.0	46.2	10.2	83.3	99.5
HAR034	cfDNA	Plasma	3.0	79.0	12.6	84.9	99.6
HAR038	cfDNA	Plasma	3.0	117.6	12.8	84.9	99.6
HAR039	cfDNA	Plasma	3.0	104.7	14.1	85.0	99.6
HAR040	cfDNA	Plasma	3.0	54.3	13.7	85.2	99.6
HAR042	cfDNA	Plasma	5.0	56.0	12.2	85.5	99.6
HAR043	cfDNA	Plasma	5.0	53.2	10.7	85.6	99.6
HAR047	cfDNA	Plasma	4.0	26.2	10.9	85.9	99.6
HAR048	cfDNA	Plasma	5.0	41.2	12.1	86.3	99.6
HAR049	cfDNA	Plasma	5.0	80.6	12.1	86.1	99.6
HAR050	cfDNA	Plasma	6.0	36.7	11.8	86.4	99.6
HAR051	cfDNA	Plasma	5.5	35.6	11.4	86.4	99.6
HAR052	cfDNA	Plasma	2.0	23.6	12.7	86.2	99.6
HAR054	cfDNA	Plasma	3.0	78.4	11.7	85.1	99.6
HAR055	cfDNA	Plasma	3.0	35.3	11.2	81.9	99.6
HAR056	cfDNA	Plasma	3.0	79.5	12.2	85.9	99.6
HAR057	cfDNA	Plasma	3.0	36.4	11.7	85.9	99.6
HAR058	cfDNA	Plasma	3.0	79.5	13.1	85.2	99.6
HAR060	cfDNA	Plasma	3.0	35.3	9.3	80.7	99.6
HAR061	cfDNA	Plasma	3.0	37.0	11.0	84.7	99.6

Patient ID	Source of DNA	Sample type	Sample volume	cfDNA amount	Depth	Mapping efficiency	Conversion efficiency
HAR063	cfDNA	Plasma	3.0	51.2	8.9	81.1	99.6
HAR064	cfDNA	Plasma	3.0	62.2	12.6	86.1	99.6
HAR065	cfDNA	Plasma	1.5	44.8	13.1	86.2	99.6
HAR066	cfDNA	Plasma	3.0	54.9	12.2	85.6	99.6
HAR069	cfDNA	Plasma	3.0	64.4	12.9	85.5	99.6
HAR070	cfDNA	Plasma	2.5	49.0	11.8	85.4	99.6
HAR071	cfDNA	Plasma	3.0	44.8	12.8	86.1	99.6
HAR072	cfDNA	Plasma	2.5	33.6	11.7	85.3	99.6
HAR073	cfDNA	Plasma	3.5	52.4	8.9	80.1	99.6
HAR074	cfDNA	Plasma	3.5	40.0	10.4	85.9	99.7
HAR075	cfDNA	Plasma	3.5	40.0	10.5	81.1	99.6
HAR076	cfDNA	Plasma	3.5	73.9	13.4	86.6	99.7
HAR078	cfDNA	Plasma	3.5	49.6	11.9	85.9	99.6
HAR079	cfDNA	Plasma	3.5	65.0	11.8	85.8	99.6

Table S4. Methylation markers in the diagnostic model.

No.	Chr.	Location	CpG number	G+C number	Genes/ lncRNA	Importance score[a]
1	chr1	237343683-237344683	16	426	*RYR2*	7.051
2	chr2	3723342-3724342	18	494	*DCDC2C*	6.856
3	chr2	3978342-3979342	5	392	NA	7.688
4	chr2	22327459-22328459	9	397	*AC096570.1*	8.380
5	chr4	164543184-164544184	6	416	NA	7.380
6	chr6	84666439-84667439	6	352	NA	7.044
7	chr8	79343444-79344444	9	396	NA	7.127
8	chr15	26569301-26570301	5	344	*GABRB3*	7.245
9	chr15	33374552-33375552	6	383	*RYR3*	6.718
10	chr15	97703143-97704143	12	409	*LINC00923*	7.643

[a] The importance scores were evaluated by the Gini index in the random forest model.

Abbreviation: No., number; Chr, chromosome; lncRNA, long non-coding RNA.

Table S5. Predictive accuracy of the cell-free DNA methylation analysis (the cfMeth score) combined with mammography and ultrasound in the discovery and validation cohorts.

	High-risk category	Low-risk category	Total	Sensitivity	Specificity	Accuracy
Discovery cohort						
Malignant	76	1	77	98.7%	68.8%	83.8%
Benign	24	53	77			
Total	100	54	154			
Malignancy rate	76.0%	1.9%	50.0%			
Validation cohort						
Malignant	22	2	24	91.7%	88.0%	89.8%
Benign	3	22	25			
Total	25	24	49			
Malignancy rate	88.0%	8.3%	49.0%			

Table S6. Breast cancer detection rate through the combined score in clinical characteristics at a specificity of 68.8%-88.0%.

	Discovery	Validation	Total
All breast cancer	98.7% (76/77)	91.7% (22/24)	97.0% (98/101)
Age			
≤ 45 yr/o	100.0% (20/20)	100.0% (5/5)	100.0% (25/25)
> 45 yr/o	98.2% (56/57)	89.5% (17/19)	96.1% (73/76)
Stage			
Stage I	96.6% (28/29)	87.5% (14/16)	93.3% (42/45)
Stage II	100.0% (28/28)	100.0% (6/6)	100.0% (34/34)
Stage III	100.0% (20/20)	100.0% (2/2)	100.0% (22/22)
Grade			
in situ	100.0% (8/8)	100.0% (1/1)	100.0% (9/9)
I	100.0% (4/4)	50.0% (1/2)	83.3% (5/6)
II	96.8% (30/31)	100.0% (14/14)	97.8% (44/45)
III	100.0% (34/34)	100.0% (6/6)	100.0% (40/40)
Molecular subtype			
Luminal	98.0% (50/51)	88.2% (15/17)	95.6% (65/68)
HER2 positive	100.0% (12/12)	100.0% (6/6)	100.0% (18/18)
TNBC	100.0% (14/14)	100.0% (1/1)	100.0% (15/15)
T			
≤2 cm	97.5% (39/40)	90.0% (18/20)	95.0% (57/60)
>2 cm	100.0% (35/35)	100.0% (4/4)	100.0% (39/39)
Lymph node			
Negative	97.6% (40/41)	88.2% (15/17)	94.8% (55/58)
Positive	100.0% (36/36)	100.0% (7/7)	100.0% (43/43)
Ki67			
≤30%	97.7% (43/44)	90.0% (18/20)	95.3% (61/64)
>30%	100% (33/33)	100.0% (4/4)	100.0% (37/37)

Abbreviation: TNBC, triple-negative breast cancer.

Reference in the supplement

1. **American College of Radiology. American College of Radiology Breast Imaging Reporting and Data System Atlas (BI-RADS Atlas). Reston, Va: American College of Radiology, 2013.**
2. McShane LM, Altman DG, Sauerbrei W, Taube SE, Gion M, Clark GM, Statistics Subcommittee of the NCIEWGoCD: **REporting recommendations for tumour MARKer prognostic studies (REMARK).** *Br J Cancer* 2005, **93:**387-391.
3. Krueger F, Andrews SR: **Bismark: a flexible aligner and methylation caller for Bisulfite-Seq applications.** *Bioinformatics* 2011, **27:**1571-1572.
4. Krueger F, Kreck B, Franke A, Andrews SR: **DNA methylome analysis using short bisulfite sequencing data.** *Nat Methods* 2012, **9:**145-151.
5. Wang K, Zhao S, Liu B, Zhang Q, Li Y, Liu J, Shen Y, Ding X, Lin J, Wu Y, et al: **Perturbations of BMP/TGF-beta and VEGF/VEGFR signalling pathways in non-syndromic sporadic brain arteriovenous malformations (BAVM).** *J Med Genet* 2018, **55:**675-684.
6. Zhao S, Zhang Y, Chen W, Li W, Wang S, Wang L, Zhao Y, Lin M, Ye Y, Lin J, et al: **Diagnostic yield and clinical impact of exome sequencing in early-onset scoliosis (EOS).** *J Med Genet* 2021, **58:**41-47.
7. Li H, Handsaker B, Wysoker A, Fennell T, Ruan J, Homer N, Marth G, Abecasis G, Durbin R, Genome Project Data Processing S: **The Sequence Alignment/Map format and SAMtools.** *Bioinformatics* 2009, **25:**2078-2079.
8. Quinlan AR, Hall IM: **BEDTools: a flexible suite of utilities for comparing genomic features.** *Bioinformatics* 2010, **26:**841-842.

9. Liu H, Liu X, Zhang S, Lv J, Li S, Shang S, Jia S, Wei Y, Wang F, Su J, et al: **Systematic identification and annotation of human methylation marks based on bisulfite sequencing methylomes reveals distinct roles of cell type-specific hypomethylation in the regulation of cell identity genes.** *Nucleic Acids Res* 2016, **44:**75-94.

10. Su J, Yan H, Wei Y, Liu H, Liu H, Wang F, Lv J, Wu Q, Zhang Y: **CpG_MPs: identification of CpG methylation patterns of genomic regions from high-throughput bisulfite sequencing data.** *Nucleic Acids Res* 2013, **41:**e4.

11. Dudoit S, Fridlyand J, Speed TP: **Comparison of discrimination methods for the classification of tumors using gene expression data.** *Journal of the American statistical association* 2002, **97:**77-87.

12. Granitto PM, Furlanello C, Biasioli F, Gasperi F: **Recursive feature elimination with random forest for PTR-MS analysis of agroindustrial products.** *Chemometrics and Intelligent Laboratory Systems* 2006, **83:**83-90.

13. Waks AG, Winer EP: **Breast cancer treatment: A review.** *JAMA* 2019, **321:**288-300.

14. Curigliano G, Burstein HJ, Winer EP, Gnant M, Dubsky P, Loibl S, Colleoni M, Regan MM, Piccart-Gebhart M, Senn HJ, et al: **De-escalating and escalating treatments for early-stage breast cancer: the St. Gallen International Expert Consensus Conference on the Primary Therapy of Early Breast Cancer 2017.** *Ann Oncol* 2017, **28:**1700-1712.

15. **American Joint Committee on Cancer (AJCC). AJCC Cancer Staging Manual. 8th ed. New York: Springer; 2017.**

16. Ostle BaM, L.C.: **Statistics in Research. Iowa State University Press.** 1988.

17. Ames IZ, Jerrold H.: **Biostatistical Analysis (Second Edition). Prentice-Hall. Englewood Cliffs, New Jersey.** 1984.

18. Hanley JA, McNeil BJ: **A method of comparing the areas under receiver operating characteristic curves derived from the same cases.** *Radiology* 1983, **148:**839-843.

19. Obuchowski NA, McClish DK: **Sample size determination for diagnostic accuracy studies involving binormal ROC curve indices.** *Stat Med* 1997, **16:**1529-1542.

20. Robinson JT, Thorvaldsdottir H, Winckler W, Guttman M, Lander ES, Getz G, Mesirov JP: **Integrative genomics viewer.** *Nat Biotechnol* 2011, **29:**24-26.
